# Security and privacy requirements for a multi-institutional cancer research data grid: an interview-based study

**DOI:** 10.1186/1472-6947-9-31

**Published:** 2009-06-15

**Authors:** Frank J Manion, Robert J Robbins, William A Weems, Rebecca S Crowley

**Affiliations:** 1 Information Science and Technology, Fox Chase Cancer Center, Philadelphia PA, USA; 2 Information Technology, Fred Hutchinson Cancer Research Center, Seattle WA, USA; 3 Academic Technology, University of Texas Health Science Center at Houston, USA; 4 Biomedical Informatics, University of Pittsburgh School of Medicine, Pittsburgh PA, USA

## Abstract

**Background:**

Data protection is important for all information systems that deal with human-subjects data. Grid-based systems – such as the cancer Biomedical Informatics Grid (caBIG) – seek to develop new mechanisms to facilitate real-time federation of cancer-relevant data sources, including sources protected under a variety of regulatory laws, such as HIPAA and 21CFR11. These systems embody new models for data sharing, and hence pose new challenges to the regulatory community, and to those who would develop or adopt them. These challenges must be understood by both systems developers and system adopters. In this paper, we describe our work collecting policy statements, expectations, and requirements from regulatory decision makers at academic cancer centers in the United States. We use these statements to examine fundamental assumptions regarding data sharing using data federations and grid computing.

**Methods:**

An interview-based study of key stakeholders from a sample of US cancer centers. Interviews were structured, and used an instrument that was developed for the purpose of this study. The instrument included a set of problem scenarios – difficult policy situations that were derived during a full-day discussion of potentially problematic issues by a set of project participants with diverse expertise. Each problem scenario included a set of open-ended questions that were designed to elucidate stakeholder opinions and concerns. Interviews were transcribed verbatim and used for both qualitative and quantitative analysis. For quantitative analysis, data was aggregated at the individual or institutional unit of analysis, depending on the specific interview question.

**Results:**

Thirty-one (31) individuals at six cancer centers were contacted to participate. Twenty-four out of thirty-one (24/31) individuals responded to our request- yielding a total response rate of 77%. Respondents included IRB directors and policy-makers, privacy and security officers, directors of offices of research, information security officers and university legal counsel. Nineteen total interviews were conducted over a period of 16 weeks. Respondents provided answers for all four scenarios (a total of 87 questions). Results were grouped by broad themes, including among others: governance, legal and financial issues, partnership agreements, de-identification, institutional technical infrastructure for security and privacy protection, training, risk management, auditing, IRB issues, and patient/subject consent.

**Conclusion:**

The findings suggest that with additional work, large scale federated sharing of data within a regulated environment is possible. A key challenge is developing suitable models for authentication and authorization practices within a federated environment. Authentication – the recognition and validation of a person's identity – is in fact a global property of such systems, while authorization – the permission to access data or resources – mimics data sharing agreements in being best served at a local level. Nine specific recommendations result from the work and are discussed in detail. These include: (1) the necessity to construct separate legal or corporate entities for governance of federated sharing initiatives on this scale; (2) consensus on the treatment of foreign and commercial partnerships; (3) the development of risk models and risk management processes; (4) development of technical infrastructure to support the credentialing process associated with research including human subjects; (5) exploring the feasibility of developing large-scale, federated honest broker approaches; (6) the development of suitable, federated identity provisioning processes to support federated authentication and authorization; (7) community development of requisite HIPAA and research ethics training modules by federation members; (8) the recognition of the need for central auditing requirements and authority, and; (9) use of two-protocol data exchange models where possible in the federation.

## Background

### caBIG and Grid Computing

An important emerging computing paradigm for life science research – grid based computing [1] – promotes large-scale sharing of data and computing resources. Grids can be classified broadly as computational grids, whose primary function is to provide large scale distributed computing capability, or data grids whose principle function is to provide the ability to query and aggregate data from multiple, independent data sources. Successful grids in both areas already exist. BIRN [2] and the @neurIST project[3] are examples of data-centric grids, while the Open Science Grid[4], and TeraGrid [5] are computationally focused grids. Launched in February 2004, the cancer Biomedical Informatics Grid [6]*(caBIG) *is a data grid under development by the National Cancer Institute (NCI) Center for Bioinformatics. As of 2007, the caBIG project included more than 1,000 individuals at over 80 institutions, including NCI-designated cancer centers, NCI community cancer centers, Clinical Trials Cooperative Groups, NCI Specialized Programs of Research Excellence, and a variety of other participants from academia and industry [7]. The goal of this effort is to provide distributed computerized systems that can speed research discoveries and improve patient outcomes by linking researchers, physicians, and patients throughout the cancer community. Federation is accomplished using advanced grid computing "middleware" based on the Globus toolkit [8] termed "caGrid" [9]. In addition to basic capabilities, such as automatic discovery of remote data services and distributed queries, caBIG seeks to provide a level of semantic inference and semantic interoperability of systems by supporting strong data typing, by providing registered models for data and metadata associated with an application, by binding data models to an underlying description-logic ontology, and through rigorous peer review during software development. A full description of the caBIG project is beyond the scope of this paper. Details may be found online [10].

### Purpose and scope of study

The purpose of this study was to develop a preliminary set of security policies and procedures applicable to institutions that participate in caBIG. To do this, the investigators used team-based methods to develop structured interview instruments and then used these instruments to systematically collect policy statements by decision makers involved in regulatory practices at six United States cancer centers. Four of the six institutions were chosen because they were in the process of adopting and deploying caTIES – a caBIG application developed by one of the authors (RC). caTIES provides a repository for deidentified data containing discrete data elements abstracted from free-text anatomic pathology reports. The caTIES application was one of the earliest grid enabled caBIG systems with the potential to share human-derived data, and thus provided a useful test-bed for discussions with stakeholders.

Although neither comprehensive nor exhaustive, this study developed valuable primary data on the attitudes of those involved in regulatory decision-making relevant to the development and functioning of a large-scale and federated, biomedical data grid. This data was ultimately used to inform development of a series of white papers [11-18] summarizing various aspects of security policy and procedural recommendations to the caBIG program office.

### Special problems posed by multi-site federations

Large-scale data sharing initiatives will be effective only if they are widely adopted. If adoption requires negotiation of specific, binding pair-wise agreements, legal or regulatory in nature, the burden of creating and managing these agreements for thousands of participants across hundreds of organizations will be the square of the number of participants, which will be prohibitive in scope and scale. Consequently, adoption models must allow regulatory needs to be met, while supporting flexibility and growth of the underlying organization. Many existing organizations have evolved in part to address this scaling issue. The cancer Cooperative Groups, BIRN and many other groups have developed reciprocal business agreements that enable linear scaling of agreements, although for clinical datasets there are typically additional agreements that are put in place that are in fact facilitated by the umbrella business agreement.

Adoption also requires trust between data providers and consumers who use the infrastructure and regulators who oversee the process. Trust relies on an understanding of the needs *all *stakeholder groups, and the development of suitable technology to meet these needs. As used in a technical context, the term "trust" describes the degree of assurance a relying party may place in a digital assertion (usually termed a "certificate") given by some entity (usually termed a Certifying Authority). These assertions may be concerned with either Authentication, i.e., who or what a given entity is, or Authorization, which deals with the rights or privileges an entity may possess. A full description of the formal concepts and foundations of trust is beyond the scope of this paper; however the interested reader is referred to the paper by Chapin [19]. An effective security system in a federated environment is well served by having a mechanism for expressing and maintaining differing degrees of this digital "trustworthiness" between multiple parties. For a description of the novel technical mechanisms developed for caBIG see the description of the GAARDS security system in Oster [9]. From a legal or governance perspective, existing federations often employ "trust agreements" of some degree to reify expectations between parties. An example of such an agreement may be seen in the InCommon Participation Agreement [20].

Regulatory personnel require that data sharing agreements and technical mechanisms used between investigators adhere to HIPAA [21], the Common Rule [22], 21CFR11 [23], and other regulations. Investigators require that the systems protect their intellectual capital. Tech-transfer officers want the system to protect intellectual property. These requirements lead to technical implications for the design, implementation, and operation of caBIG systems including how potential users at multiple sites are identified, made known to, and ultimately authorized to access those systems.

From its inception, the caBIG project has been committed to a federated, as opposed to a centralized model. In this federated model, data are stored and managed locally in systems that can communicate with other geographically distributed systems using the capabilities of the caGrid middleware. In principle, each individual research group or institution can retain ultimate control over who has access to its data at all times. However, accurate access-control (i.e. authorization) decisions cannot occur without knowledge of who is requesting access, for what purpose, and with what authority. Consequently, caBIG includes identity management processes in its federation model to provide the needed authentication on which authorization decisions ultimately rely.

If caBIG or any federated biomedical data grid is to meet the needs of all relevant parties, those needs must be known – especially those of the often non-technical staff charged with overseeing data integrity and privacy.

### Existing Regulatory Constraints

There are several regulations that must be recognized and addressed for federated biomedical grids such as caBIG to function effectively. The following regulations are not intended to constitute an exclusive list of all potential regulations affecting biomedical grids, as there are numerous federal and state regulations that will affect operations. Below, we list and briefly introduce the key regulations governing federated biomedical data sharing consortia.

#### HIPAA

The Health Insurance Portability and Accountability Act[21] Privacy Rule found in 45 CFR 164, regulates the use and disclosure of Protected Health Information (PHI), including PHI's electronic transmission. HIPAA imposes important requirements for research performed using caGrid, including strict requirements for informed consent and data de-identification.

#### Institutional Review Boards

Institutional Review Boards (IRB) have the authority to approve, require modifications, or disapprove and disallow research on human subjects under Food and Drug (FDA) and Health and Human Services (HHS) regulations[15]. IRBs may require institutions to implement specific IRB and HIPAA training programs and other policies and procedures for institutions and researchers to perform human subjects' research. For institutions to obtain IRB approval to participate in caGrid, it appears IRBs may seek reassurance of the ability of caBIG to ensure safe practices for human subject research by all caGrid participants, including compliance with honest broker and informed consent requirements.

#### IACUC

The Institutional Animal Care and Use Committees provide regulatory oversight of research involving laboratory animals. Every institution that uses animals for federally funded laboratory research must have an IACUC, which reviews research protocols and evaluates an institution's animal care and use.

#### 21 CFR Part 11: Electronic Records and Signatures

21 CFR 11 consists of FDA regulations for electronic records and electronic signatures to be considered trustworthy and equivalent to paper records and handwritten signatures. Part 11 requires various controls, including audits and validation systems, to be implemented as part of a regulated entity's operations.

#### Federal Employee Regulations and Standards

There are various federal regulations and standards governing federal employees' and contractors' use of electronic equipment, such as the Federal Information Processing Standards 201–1 (Personal Identity Verification requirements), that will have some impact on caBIG.

#### State Privacy Laws

Each state may establish its own privacy laws, governing the use and disclosure of personal information. These laws vary by state, and may be more stringent than federal laws, such as HIPAA, requiring additional regulatory compliance by institutions in those states.

### The Structural Basis of Federations

Federations by definition consist of multiple entities which must be bound together by a shared framework of governance. The Liberty Alliance [24], a consortium working to define interoperable federated computing environments, defines three major governance models for federations [25]. Each model has specific strengths and weaknesses. These constraints must be understood in selecting a governance model and developing policy. To operate, federations typically must have agreements in place to describe the structure of the federation, how it will be governed, the requirements and rules expected between the various parties. Consequently, establishing a federation requires higher level governing structures, guidelines, and policies. These are in addition to the security, privacy, and data sharing policies of the individual organizations. Since trust relies on the adherence to agreed upon policies in these areas by all participants, some degree of policy reconciliation between the members of the federation is usually necessary. Three pertinent examples of moderately mature federated environments are presented below.

#### Liberty Alliance

The Liberty Alliance [24] is a group of over 30 commercial and other organizations formed to establish open standards, guidelines, and best practices for federated identity management. The group has been a leader in the specification, certification, and development of various protocols, guideline documents, and policies related to developing successful wide-scale identity federations.

#### Safe-BioPharma Association

The Safe-BioPharma Association [26] is a group that has developed and promoted specific digital identity and digital signature standards to promote interoperability of systems across corporate boundaries. As such, they function as a federation. The federation focuses on the specific business requirements and interchange of information between the BioPharma industry, various regulatory bodies, such as the FDA, and the healthcare industry.

#### InCommon

InCommon [27] is an identity federation run by a large consortium of institutions of higher education in the United States. The goal of the federation is to promote interoperability of systems across institutional boundaries for faculty, researchers, staff, and students in the US research and education sphere. As of October 2008 the consortium lists over 2.2 million users in over 108 academic and research organizations, and it includes major academic publishers, libraries, 72 higher education participants, including a number of large state university systems, and several major government and government-sponsored programs. Of particular relevance for this paper are the NIH and TeraGrid.

## Methods

Our approach was to develop structured elicitation interviews of key regulatory personnel at a subset of cancer centers involved in exchange of data using the caBIG system. Interview instruments were developed using a team-based approach. Regulatory participants were recruited, and telephone or in-person interviews were conducted. Results were tabulated according to job description, type of institution, and other relevant classifications. These were used by the investigators to determine the stated fundamental security and privacy drivers involved in a multi-center use of the grid for de-identified data exchange.

### Development of the interview instrument

The interviews utilized problem scenarios developed collaboratively during a one-and-a-half day intensive face-to-face meeting that occurred in Pittsburgh, June 12–13, 2006. Thirty-eight individuals representing a wide spectrum of experts and stakeholders from US Cancer Centers and the NIH spent approximately four hours discussing and brainstorming about potential barriers to the multi-institutional sharing of data, through caBIG. Individuals who participated in the development of the instruments included representatives of the security project (7), members of the Data Sharing and Intellectual Capital Working Group (3) and Architecture Working Group (1), Institutional Review Board directors (3), external advisors (3), grid technologists (3), NCICB representatives (5), patient advocates (3), caTIES adopters (4), caTIES development team (2), and other stakeholders (4).

Meeting participants were asked to think broadly about issues that might pose problems, particularly those where we expected significant variation among cancer centers. Issues were collected into a master list and sorted into four general categories. The categories which emerged from this process were: (1) Locus of control/decision making, (2) De-identification and IRB Policy, (3) Authentication and Authorization, and (4) Consenting.

Participants then divided into four breakout groups, one for each of these major themes, and constructed scenarios and draft interview questions designed to elicit information during the interviews. All scenarios used caTIES as the example system. Participants met at the end of the day to critique the resulting scenarios.

Following the face-to-face meeting, the authors edited the interview scenarios to ensure adequate coverage of the issues, improve the understandability and simplicity of the interview questions, and match interview questions to organizational roles of interviewees. The resulting draft instruments (see additional file 1) were reviewed by all meeting participants, and modified in three subsequent rounds of editing and draft revisions. Together, the four interview instruments contained a total of 87 questions. The topic of each scenario along with the organizational roles of intended respondents and the number of questions are shown in Table 1.

**Table 1:** Questions intended for each type interviewee.

Scenario	Topic	Target respondents	Questions
1	Identification of local, organizational environment, stakeholders and decision-making processes	IRB members, security officers, HIPAA compliance officers, Office of Research administrators	1.1 – 1.19

2	De-identification	IRB members	2.1 – 2.22

3	Auditing	Security officers, HIPAA compliance officers, Office of Research administrators	3.1 – 3.20

4	Prospective Research Consenting	IRB members, Office of Research administrators	4.1 – 4.9

### Participants

We contacted individuals across six United States (US) cancer centers involved in the caBIG project. Participating cancer centers included all four current adopters of the caTIES System, the test-bed system described in the Interview Instrument. All four are university-affiliated. Two other institutions represented stand-alone cancer centers involved in the caBIG project, and were included to broaden the sample, because of a concern that data obtained from the four university-affiliated cancer centers might not generalize to stand-alone cancer centers. These two centers represented a convenience sample of centers affiliated with the authors. The total percentage of standalone centers in this sample (2/6) is similar to the percentage of stand-alone cancer centers across the nation (13/63).

For each institution, we asked a collaborator at that institution to identify key individuals with decision-making authority who, we anticipated, would need to be involved in the development of a federated grid for data sharing across institutions. The roles of these individuals thus varied somewhat based on the organizational structure and culture of the participating institution.

### Data Collection

Interviews were conducted either on-site (N = 5) or by telephone (N = 14), based upon conditions of approval of the participating institution. For all interviews, we provided participants with the interview scenarios in advance. Interviews were recorded as digital files, and transcribed verbatim. The interviewer maintained a key indicating the organization and role of the participant. Identifying information regarding participant and institution was scrubbed from the resulting documents to generate the final de-identified transcripts.

### Data Analysis

The interviewer manually coded the interviews, using principles of both quantitative and qualitative data analysis.

#### Quantitative Analysis

The interview scenarios were structured such that individual participants were asked a subset of the 87 questions across four scenarios, based on organizational role and expertise. Responses to the 87 interview questions were aggregated in Excel. For some objective questions regarding organizational policy or processes, only a single answer was sought from an individual with sufficient authority to respond. Consequently, during the analysis phase, we chose to alternate the unit of analysis depending on the interview question.

For questions related primarily to the institution, we aggregate all information from multiple individuals across a single institution and present statistics with *institution *as the unit of analysis. For questions where each participant provided a single response, we show counts with *interview *as the unit of analysis. When two individuals were interviewed together, and we found no instances of disagreements, we recorded only one response per interview. For questions where participants enumerated multiple items in response to a question, we use *interview statements *as the unit of analysis.

#### Qualitative Analysis

Many issues were discussed during these semi-structured interviews that provide guidance for developing security processes and policies. Key issues and opinions from all interviews were highlighted in the files, and used to distill a set of themes and issues for qualitative data analysis. Areas where there appears to be consensus and areas that show strongly divergent views are discussed using quotations from the primary data. Commonly accepted editing and proofing standards were used to clarify quotes when necessary without changing the contextual meaning. For example, any added words or phrases appear in block parenthesis []. Every precaution was taken to maintain the integrity of the original quotes. In order to assure that quotations were representative of the entire sample and not a small set of participants, we examined the distribution of quotations after the manuscript was completed.

## Results

### Characteristics of the Interview Participant Sample

We contacted thirty-one (31) individuals at six cancer centers with requests to participate. Twenty-four out of thirty-one (24/31) individuals responded to our request- yielding a total response rate of 77%. The distribution of organizational affiliation of participants is shown in Table 2. Nineteen total interviews were conducted over a period of 16 weeks.

**Table 2:** Institutional Affiliations of Interview Participants.

Institution	Cancer Center Organizational Structure	caTIES Adopter	Number of Participants
A	University Affiliated	Yes	4

B	University Affiliated	Yes	7

C	University Affiliated	Yes	3

D	University Affiliated	Yes	1

E	Stand-Alone	No	4

F	Stand-Alone	No	5

Total			24

At one institution (Institution D), we were only able to recruit a single participant. Therefore, for questions in which the unit of analysis is the institution, we include only five of the six institutions. Data obtained from the single individual from cancer center D is included only in quantitative analyses where the unit of analysis is the individual and in qualitative analyses.

Fourteen interviews were conducted with one participant only and five interviews were conducted with two participants together. In all interviews where two participants were interviewed together, the pairs consisted of supervisor-supervisee dyads that worked at the same institution. In all cases, one of the two individuals originally contacted specifically requested that their supervisor or supervisee participate jointly in the interview.

The roles of participants within their organizations are shown in Table 3. In some cases, individuals served in multiple capacities within their organizations (for example information security officer and privacy officer); therefore, the total number of roles recorded in Table 3 exceeds the number of respondents.

**Table 3:** Organizational Roles of Participants Interviewed.

Organizational Role	Count
University and IRB legal counsel	3

IRB Director or Chair, or Director of Human Subjects Protection	5

IRB Regulatory Affairs Officer	1

Information Security Officer	3

Hospital Privacy Officer	3

Hospital Compliance Officer	1

University or Research Institution Privacy Officer (supervising Hospital Privacy Officer)	4

University or Research Institution Compliance Officer	3

Institutional Strategic Planning Executive	2

Director of Office of Research, or Vice President for Research	3

Hospital Department Director of Information Services	1

### Analysis of interview responses grouped by theme

**Figure 1:**
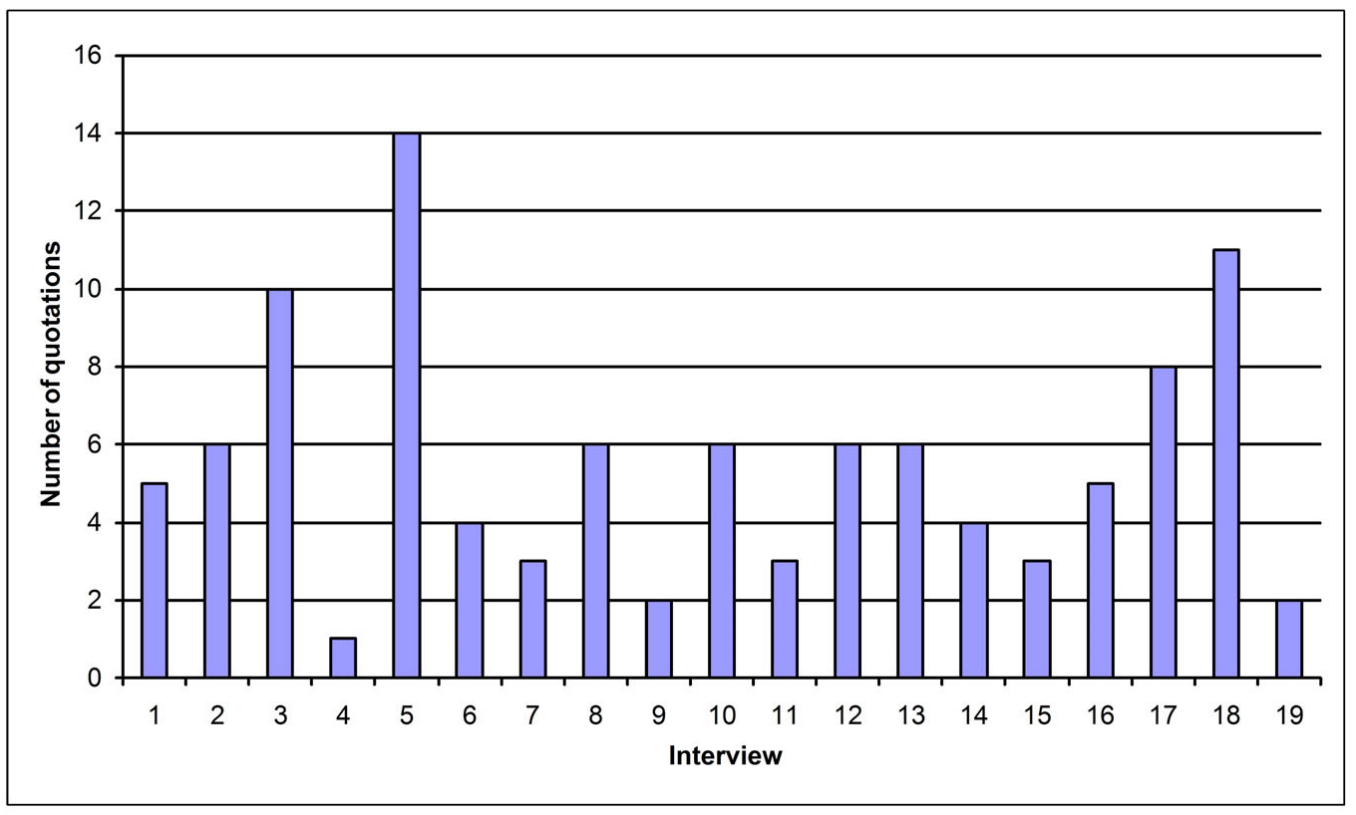
**Distribution of quotes across the participants**.

The following sections contain responses to the interview questions grouped by theme, and include both quantitative and qualitative analyses of the pattern of responses. Tables indicating quantitative results include captions which describe the total number of respondents and their organizational roles. Questions posed in each interview were specific to organizational role, and hence the denominator varies with each question. In addition to aggregating and quantifying the responses, we also looked for issues or requirements that could have technical, as well as policy or procedural, implications for the operation of caGrid. Figure 1 depicts the distribution of quotations across interviews, and shows that all participants are represented in the analysis.

### Project structure and governance

#### Necessity of a governance structure

Over 85% of individuals expressed the opinion that multi-institutional data sharing through the caGrid requires a governing body (Table 4).

**Table 4:** Does the caBIG project require a governing body for data sharing?

Response	Count	Percentage
Yes	14	87.5

No	1	6.3

Unsure	1	6.3

Scenario 1 – Question 3. A total of 16 interviews provided responses. Respondents included individuals from all organizational roles. Data was aggregated with interview as the unit of analysis.

The need for a governing body was expressed across the entire spectrum of organizational roles, from IRB directors to information technology (IT) security managers to privacy officers and Office of Research representatives:

"I do think that from an institutional level there should be a governing body to provide guidance and to enforce policy, and to make policy for all the systems that will interact and handle activity with other institutions. As far as what functions they would dictate, [they] would be all around the authorization, authentication, and accounting of access to that data."

- IT Security Manager

*I personally think there needs to be a governing body. I know there is a great desire not to have any type of centralized functions ...but I think there needs to be [to] provide an audit and oversight function capability. It also has to provide the process by which people become certified to receive data, and I think also it needs to make decisions when somebody is no longer entitled to receive data, whether that be because they are no longer part of the project or that . . or in some way jeopardized their standing due to having done the wrong thing with the data, but I think there needs to be that level of oversight*.

- Privacy Officer

Others felt that a governing body would be useful but that it was critical to achieve the right balance between guidance and standards at the multi-institutional level, and the flexibility to interpret and adapt them at the local level:

"I think operations are to be [at] the local level and standards at the network level, if you will, so similar to IRB standards or rules, those are set at a federal level but administered locally. And, there is a process for auditing as to whether or not the standards are met, and I think that builds in the most consistency at the one level, because you do not want people engaging in different practices, and the most flexibility at the local level... I think the major point of making good general rules, good general standards, using standards that are already out there, and then letting institutions administer them locally is the best... I used to be VP for Regulatory Affairs, and I cannot think of a regulation that I would like to have administered from on high, because you just cannot know the local circumstances."

- Vice President for Planning and Business Administration

#### Potential functions of a governing body

Participants suggested a large number of potential functions for the governing body in overseeing the sharing of data. All responses collected are enumerated in Table 5.

**Table 5:** Potential functions of the governing body suggested by participants.

Functions of a Governing Body Suggested by Interview Participants	Count
**Data Use**	

Establish principles of operation of the community	3

Make project-wide decisions regarding appropriate use of data and tissue (rules of engagement)	5

Establish uniform position on data ownership and intellectual property	1

Set standards for assuring data integrity	1

Establish common guidelines on professional credentials needed to access specific types of data	2

Oversee the "joining" of organizations	4

Review privacy laws and research ethics guidelines for potential foreign partners before entry	2

**Community-Wide IRB Functions**	

Provide community-wide assurance that all repositories have appropriate IRB review	1

Establish common Data Safety Monitoring Plans agreeable to constituent IRBs	1

Act as a community-wide Data Safety Monitoring Board	1

Establish standards for Human Subjects Research (HSR) and HIPAA training; require institutions to assess own training modules; publish results to community	1

Provide guidance on common consent form language across caBIG	2

Random checks of user publications to determine whether data use appropriate to protocol	1

**Risk Assessment**	

Establish common levels of data risk and identify security mechanisms appropriate for risk level	1

Provide centralized statistical assurance of minimal risk of re-identification for systems	2

**Establish Security Policies and Processes**	

Prevent and police abuse	4

Establish common guidelines for provisioning and de-provisioning users	2

Establish requirements for monitoring credentialing process and assess incoming progress reports	2

Establish standards for authorization	2

Set minimum standards for physical security	2

Set standards for what users will have to agree to do and not do	1

**Audit and Oversight**	

Aggregate audit information and provide reports back to member institutions	2

Monitor compliance with established and agreed upon processes	2

Periodic checks of whether the data which is supposed to be de-identified is REALLY de-identified	1

Investigation of security incidents	1

**Reporting and Enforcement**	

Establish enforcement policy for sanctioning of organizations or individuals who misuse resource	1

Report misuse to OHRP, ORI and funding agency when necessary	1

Issue federation-wide reports of security incidents	1

Maintain federation "No Fly" list of researchers not permitted access anymore from any institution	2

**Mediation**	

Mediate disputes between organizations	2

Accept requests to appeal decisions at local institutions (for example termination of access)	1

**Build Trust within the Community**	

Build trust among institutions that data will be used appropriately	3

Build trust in veracity of user identities	1

**External Standards and Best Practices**	

Set external standards participating institutions must meet (e.g. CLIA approval of tissue-banks)	1

Seek out and publicize community-wide best practices	1

**Strategic Role**	

Establish goals for the entire project and ensure that operation is in keeping with those goals	1

Monitor new regulations coming from the federal government and address relevance to sites	1

Assess and address weaknesses of the collaborative research environment	1

Address novel problems	1

Scenario 1 – Question 3. A total of 17 interviews provided responses. Respondents included individuals from all organizational roles. Data was aggregated with interview statement as the unit of analysis

The resulting functions cover a broad range of categories including common guidelines for data use, community-wide IRB functions, risk assessment, general security policies and procedures, audit and oversight, reporting and enforcement, and selection of external standards for operation. In addition to the operational functions, participants also suggested several more abstract responsibilities. Some participants indicated that the governing body was necessary in order to build trust among the participant organizations. Participants also suggested that the governing body must provide a strategic role, for example by monitoring the Office of Human Research Protections (OHRP) regulations or new laws that might affect the use of the federated grid.

#### Requirement that the collaboration be a legal entity

One university legal counsel articulated the need for the collaboration to be a legal entity. The benefit of a legal entity is that the entity carries insurance and provides a single point of authority for enforcement should the terms of the contract be breached. The legal entity reduces risk to individual participating organizations.

"Is this going to be an incorporated entity? Because that is going to be big, because when you talk...to lawyers about this, if there were for example, an institution [that] said that we are going to be the people who are responsible for administering all of this and signing the contract. I can sign an agreement with them in which they agree to handle my data a certain way, and I agree to make the data available to them for approved users. I know them. They have insurance. They have lawyers who make sure that...they will go after people if something is violated ... I can go to them and say you've breached your agreement because this person has done this and they will go after them. If you are talking about it being open, a consortium of entities with just an MOU (Memorandum of Understanding) or something in place that's not going to work"

- University and IRB Legal Counsel

The need for a legal entity was posited regardless of whether data was identified or de-identified. This participant suggested that caBIG consider forming its own non-profit incorporated entity. The formation of such an entity would greatly simplify the legal requirements for joining caBIG for this institution. In fact, the institution has previous experience with data sharing under these conditions:

"I actually just had one the other day where the entity that is incorporated . . .where you can see this sometimes is...the incorporated non-profit [consists of] institutions that want to share resources or want to come together to facilitate research on a specific disease... often a kind of a rare organ disease. So I just got something from another institution where there is this network of doctors who are all interested in research on this rare disease, and in that case, they actually formed a separate non-profit that is functioning like a contract research organization for that disease"

- University and IRB Legal Counsel

In the absence of an incorporated entity, this participant suggested that it would be necessary for the institution to sign separate Data Use and Confidentiality Agreements with each participating organization. To streamline the process, the participant suggested using common forms for Data Use and Confidentiality Agreements between institutions and Authorized User Agreements among users. The Data Use agreement may need to specify that the receiving organization is responsible for policing compliance. Institutions may need to understand exactly what resources are necessary for meeting these compliance requirements.

### Trust agreements

Participants recognized the importance of agreements between institutions and were largely in agreement with what such documents should contain.

#### Important areas to be covered under trust agreements

The majority of participants agreed that documents should contain language related to all of the elements described in Table 6.

**Table 6:** Which elements should be included in the trust agreements?

Element of Trust Agreement	Yes		No		Unsure	
	**Count**	**%**	**Count**	**%**	**Count**	**%**

Integrity Protections	7	87.5	0	0	1	12.5

Assurance that staff will receive training including on privacy and security	8	88.9	1	11.1	0	0.0

Agreement to participate in defined security incident response policies	9	100.0	0	0.0	0	0.0

Statements that users will not re-identify	10	100.0	0	0.0	0	0.0

Statements that users will not use data for any other purpose	10	100.0	0	0.0	0	0.0

Liability Allocation	8	80.0	1	10.0	1	10.0

Indemnification	5	71.4	1	14.3	1	14.3

Penalties for breaching terms of agreement	8	88.9	1	11.1	0	0.0

Scenario 2 – Question 16. A total of 10 interviews provided responses. However not all respondents considered themselves sufficiently expert to determine the importance of inclusion of specific items, hence the denominator of the table varies by item between 7 and 10. Respondents included university and IRB legal counsels, IRB directors, Office of Research representatives, and Privacy and Compliance officers. Data was aggregated with interview statement as the unit of analysis.

We also asked participants to suggest other potential areas that should be covered in the trust agreements. Areas suggested included language on intellectual property, agreements to participate in a compliance program including audits, agreements to be bound by the local IRB, and statements that data is not provided with a warranty of compliance (Table 7).

**Table 7:** Other elements that should be included in trust agreements.

Additional Suggested Elements of Trust Agreements	Count
Agreement to participate in compliance program including audits	4

Intellectual Property	2

Statement that repositories will be IRB approved, and that users will abide by IRB practices	2

Statement that data is not provided with warranty of compliance	1

Scenario 2 – Question 17. Data was aggregated with interview statement as the unit of analysis.

#### Indemnification and liability allocation

One participant indicated that their institution typically included a statement that the institution providing data made no warranty of its compliance. This requirement that institutions be able to submit data with no warranty as to their compliance status is completely contradictory to another requirement that "local caBIG repository owners and stewards need to be able to define and attest to the risk level specific to their context and state law. Sharing of data must operate under these constraints." Additional work is needed to determine how best to reconcile these opposing positions.

Assuming for the moment, that caBIG does try to support warranty-free data sharing, it may be difficult to get all institutions to agree to a blanket, use-at-your-own-risk policy. However, one interviewee noted that a more general statement about responsibility for acts of negligence might meet with less resistance:

"Typically what we would do is we would state that the data is not provided with any warranty with respect to its suitability or with respect to its compliance. The receiving entity is going to want to take responsibility if there was a mistake in the de-identification process, and data gets out. We are going to want them to assume liability for anything that happens to the data once they get it. You're right. State institutions will not agree to this indemnity provision. I think we might be able to come up with a statement that is very benign that says that each party is responsible for acts arising from their own negligence...Universities collaborate with each other all the time. We call them subcontracts on federal grants, and then we both fight over indemnification because the federal demonstration project, which was a project to try to come up with a common form for subbing federal grants, takes the approach that sort of we all say 'Everybody – you are responsible for what you do, I am responsible for what I do.' End of story. And I would suggest that you take a similar approach in this kind of agreement."

- University and IRB Legal Counsel

Another participant noted that although liability and indemnification were useful legal tools, they did not address all kinds of risk:

"From a risk perspective, there are different kinds of risks... financial risk, operational risk, reputation risk, compliance risk... the liability and indemnification... minimize financial risk and maybe operation... but they do not eliminate reputation risks, and that could be the biggest risk, especially in research where people may shut down your project."

- University Privacy Officer

From this participant's perspective, agreements could only go so far. Other protections, such as auditing and compliance checking, may well be necessary to minimize more substantial risks such as those to reputation.

#### Intellectual property

Participants differed markedly as to whether language regarding Intellectual Property (IP) should be included in the agreements. Many participants felt that the IP belonged solely to the individual making the discovery, and that each organization had an equal opportunity to gain from the aggregated data, leaving it to them to exploit this advantage:

"Because the data would remain the property of the providing party, and so [in] the agreement you would really have to specify that the data remains the property of the providing party – they are only getting the right to use it. Once somebody does use the data, in my opinion, the intellectual property would be owned by the person who made the breakthrough."

- University and IRB Legal Counsel

"But I don't see this stuff as anything but raw material. The inventor's act takes at (sic) place at the receiving institution, and if that's the case, the providing institution has no role in the invention whatsoever. It's a raw material... It's the hammer and nails. You make a house of it, it's yours... If you really know you have the most precious nail there is, then you have a couple of choices. One is do not put [it] in the repository in the first place. Number two is make it available with some level of restrictions, through an NDA, like for research purposes only. And number three, is give it away freely. Those are your only choices. And none of those require a complicated legal agreement."

- Vice President for Planning and Business Development

Other participants were not as willing to make such a clear distinction between the inventor and the provider of the information, or materials used in the invention, especially if they retained ownership of data throughout its use:

"I would think that the only thing you could really stipulate by contract up front would be that there...in the event of a discovery, all of the parties need to be informed, and they would cooperate in coming up with some allocations. Another way to think about it is what's happening with the data ownership? If we are going to say – great we are participating, and the moment it becomes de-identified, it is no longer our data, but part of the collective data, and then the invention, any intellectual property rather that is a result of any use of that data will reside where the person is employed. If, however, the ownership is not sort of given up into this collection, that's going to be much more problematic."

- Director, Officer of Research Administration

Another concern raised about intellectual property was the potential for data to become available through the grid inadvertently that is owned by some third party, potentially a for-profit entity:

"I'll tell you the thing that worries me more than that is valuable information or samples that have been obtained from commercial parties under NDAs put into the tissue repositories without any markings on them whatsoever..."

- Vice President for Planning and Business Development

Further discussion of Intellectual Property considerations are addressed in an associated white paper produced by the authors for caBIG [12].

#### Authorized user agreement

One university has an existing project with some parallels to the caBIG. The project aggregates public health data, and makes it available to institutions including public health departments throughout the country. The project has developed an authorized user agreement that users at the external institutions must sign as part of the process of establishing access.

"What we do is we sign the agreements that get the data in from both the commercial organization and hospitals. We aggregate it here, then we sign agreements with each health system that wants to access the data in which they agree to use the data only for certain purposes. They acknowledge in writing that we get the data under confidentiality restrictions, and they agree that anybody who is going to access it from the public health system has to sign what we call an authorized user agreement... a one-page agreement that states... that they are only accessing it for their job.. . they are not going to do anything else with it."

- University and IRB Legal Counsel

The need for users to agree to attest to their agreement to abide by particular safeguards was echoed by a number of participants:

"And a legal agreement that each individual agrees to abide by when they ask for access . . .not just the institution, it's the individual."

- Health System Privacy Officer

#### Data Ownership vs Stewardship

Most participants did not appreciate a difference between stewards and owners. Others had definitions that were not in agreement with the interviewer's definition. In retrospect, the answers to this question would have been more informative had the interviewer provided some definition regarding these terms.

### Scope of the collaboration

#### Effect of joining of new organizations on IRB processes

An important finding of these interviews is that many participants will be willing to accept that individual organizations may join the community, without explicit approval of every other institution (Table 8). As long as new organizations agree to abide by the same principles, the addition of a new institution appears to pose few specific barriers.

**Table 8:** Would the joining of a new organization pose a specific problem?

Response	Count	Percentage
Yes	0	0.0

No	7	100.0

Scenario 2 – Question 14. A total of 7 interviews provided responses. Respondents included university and IRB legal counsels and IRB directors. Data was aggregated with interview as the unit of analysis.

IRBs would find it useful to have an online registry, which displays all organizations that have signed agreements:

"If there is a new institution coming in, we would like some kind of registry process... maybe it could be just something that is done online, and you can look up and say 'okay. M.D. Anderson just signed on.' I guess that's okay."

- IRB Director

However, some participants were concerned that joining organizations must be able to demonstrate that they have sufficient resources and sophistication to implement both the security technology as well as the security processes. The problem could become that we are all only as strong as our weakest link:

"Even if we all had common agreements, not all cancer centers that are in caBIG have the same level of sophistication of some of the academic medical centers. Even if you gave them a contract and told them to use them, there would be concerns that there might not be the same resources to ensure appropriate implementation if it were distributed... and again, I assume there are no dollars associated with that about setting up a system that is going to require dollars to manage and maintain over time, and your institutions... you have the responsibilities in the form to do it but you do not necessarily have the dollars or the right incentives to ensure they do it right. At this place $600-million in sponsored research a year, we have got resources to ensure compliance. Not all places do that. And that would be my concern."

- University and IRB Legal Counsel

These concerns highlight the need for a process of credentialing institutions that will participate in the data-sharing community.

#### Foreign partnerships

There were significant concerns about the inclusion of foreign partners (Table 9), for a variety of reasons.

**Table 9:** Concerns described by participants regarding foreign partnerships.

Specific Concerns	Count
Privacy requirements are different than US	4

Contracts are difficult to enforce overseas	2

Concerns about potential national security threat	2

Quality of foreign partner IRB review varies greatly	2

Cannot ensure that foreign partner will not be violating their own laws	1

Increased security may be necessary	1

Research ethics guidelines vary greatly	1

Scenario 2 – Question 22. A total of 9 interviews provided responses. Respondents included university and IRB legal counsel, IRB directors, office of Research representatives, and Privacy and Compliance officers. Data was aggregated with interview statement as the unit of analysis.

Although few participants would preclude foreign partnerships (Table 10), many wanted additional assurances and controls.

**Table 10:** What do you want to see from a foreign partner?

Response	Count	Percentage
Would not want foreign partner	1	8.3

Concerns about foreign partnerships may necessitate additional requirements	9	75.0

No specific concerns as long as partner meets same standards as US partner	2	16.7

Scenario 2 – Question 21. A total of 12 interviews provided responses. Respondents included university and IRB legal counsels, IRB directors, Office of Research representatives, and Privacy and Compliance officers. Data was aggregated with interview as the unit of analysis.

Some participants were pessimistic about the inclusion of foreign partners given the wide gap in policies:

"We would not deal with Europe. They have too hard of a standard... caBIG has to involve international partners, but they also have to make sure that it is realistic to do so, given that each culture or country or union (like EU) has their own unique regulations about electronic data transfer issues in research."

- IRB Director

#### Commercial entities as partners

Several participants considered use of caBIG data by commercial entities as problematic. The concern was that commercial entities could exploit data for purposes other than the advancement of science. In particular, there was a concern that data might be passed on to commercial entities without the knowledge of the providing institution:

"I want to be sure they are not marketing... that once they get this data, that there are restrictions on them passing it on."

- University Compliance Officer and IRB Legal Counsel

The potential of commercial entities to gain access to data was considered problematic by one participant because of issues related to private inurement. Private inurement – the benefit of a private interest at the expense of the non-profit – is prohibited under law.

"When we are dealing with private industry, from my perspective, there is potentially a private inurement issue here. If somebody in industry gets our data and uses it for some type of financial gain to that company. In theory, private inurement of a non-profit organization means it cannot give something of value and not get something in return... The concept is (that) a nonprofit institution would violate it's nonprofit status by providing something of value to a for-profit company. You have to get value for value. Because otherwise, I'm giving away something which I have... something of value for which I have... which frustrates my not-for-profit status or purpose, and if you are willing to give away things like that, then I guess the argument goes that there is no need for you to be a not-for-profit at that point in time."

- Health System Privacy Officer

### Existing organizational infrastructure for data sharing

The development of the envisioned research grid will need to rely on local institutions to implement the processes in addition to the software. We found significant variation in the infrastructure existing at these organizations that could support federated data sharing.

#### Existing honest broker systems

Honest broker systems (also referred to as trusted third parties) have been developed at some institutions to provide de-identified data, compliant with the requirements of HIPAA "safe harbor" [28]. The "honest broker" acts as a trusted, neutral third-party, often regulated by the IRB, and may maintain the key which links the de-identified record and the original identifier. Although this method has been used locally, there have been no previous attempts to deploy such a system across a federated grid.

#### Description of existing honest broker systems

Only one institution indicated that they had a formal human honest brokering system in place, which was established and monitored by the Institutional Review Board (Table 11).

**Table 11:** Institutions with Human Honest Broker Processes

Existing Honest Broker Human Systems	Count
Institutions with formal process	1

Institutions with informal process	2

Institutions without any identifiable process	2

Scenario 1 – Question 1. A total of 16 interviews provided responses, from 5 institutions. Respondents included individuals from all organizational roles. Data was aggregated with institution as the unit of analysis. For responses, where there was disagreement, we accepted any description of an existing informal process by any individual at that institution as evidence of an existing informal process.

"The way we have the honest broker system set up is that the healthcare organization certifies honest brokers, and those honest brokers are typically at the department level. It's not only on a particular projection or research project by project basis, and so once those honest brokers have been certification [sic]) by a certification process involving ultimate sign off by the IRB as well as the privacy officer, once certified, then those honest brokers would work either at the department level or project level to take data from health organizations and to de-identify it for the use by an individual research project."

- Privacy Officer

Other participants described less formalized systems that had developed over time where specific individuals had the capability to de-identify data and this mechanism began to be used by outside investigators:

"I don't think we have a true honest broker system. What we have is an individual or a group of individuals who will consult and will actually provide the mechanisms for de-identifying data when asked."

- IRB Director

Other institutions had no existing mechanism to provide such a disinterested party and opportunity for maintaining a linkage file, which would permit re-identification to the disinterested party but not to the investigators.

"I don't even think there is probably a disinterested party that ever has done this either... if they de-identify, it would be... one of the people in the research team that would do it."

- University Compliance Officer and IRB Legal Counsel

Participants who did not have any kind of honest broker system nevertheless recognized the potential of such a system to enhance the functioning of a data-sharing grid:

"I like the idea of this disinterested person being able to re-identify, but again, under very controlled circumstances."

- University Privacy Officer

The main benefit of such an arrangement appears to be the potential to keep data identified only at the source institution. The additional IRB requirements that may be necessary for federated sharing of re-identifiable information, suggests that the community should study whether honest broker systems could reduce the number of cases where identifiable information is necessary.

#### Existing approved process for automated de-identification

Two of the five institutions had experience with using an automated method for text de-identification. One of these two institutions has a formal policy regarding text de-identification, which stated that data that had been scrubbed by a specific system could be considered to be "de-identified".

#### Re-identification

All participants indicated that when using a disinterested party (honest broker), it was an acceptable practice for the disinterested party to maintain a linkage file in order to allow for re-identification of the patient or participant by the disinterested party for the purpose of including additional data, as long as data remained de-identified to the investigator (Table 12). The use of a disinterested party and maintenance of a linkage file are described in the HIPAA regulations.

**Table 12:** Can data be re-identified under an Honest Broker system?

Response	Count	Percentage
Yes	12	100

No	0	0

Scenario 1 – Question 2. A total of 12 interviews provided responses. Respondents included individuals from all organizational roles. Data was aggregated with interview as the unit of analysis.

### Existing organizational decision-making structure related to privacy

We also found marked variation in the organizational infrastructure underlying decision-making in the area of privacy (Table 13).

**Table 13:** Who interprets HIPAA regulations at your institution?

Response	Count	Percentage
Privacy or Compliance Officer with IRB	3	21.4

IRB in conjunction with Legal Counsel	1	7.1

Compliance Officer with University Counsel	2	14.3

IRB or IRB Privacy Board	2	14.3

Privacy Officer	4	28.6

Formal mechanism being defined	1	7.1

Not Applicable – Not a covered entity	1	7.1

Scenario 2 – Question 11. A total of 14 interviews provided responses. Respondents included individuals from all organizational roles. Data was aggregated with interview as the unit of analysis.

Participants identified a wide range of organizational structures regarding decision-making about privacy policy and the interpretation of the HIPAA. The determining factor appears to be the relationship of the medical school or university to the health system or hospital, producing a wide variety of configurations:

"We have a HIPAA privacy officer for health systems, a HIPAA privacy officer for research and a HIPAA privacy officer in her legal office, and then one at a university level that is sort of a king/queen HIPAA privacy officer over all the other officers ... so that's kind of a funny model. So the health system has a privacy officer who is in charge of managing all disclosures whether they be research or health care."

- Director, Office of Human Research

"We do have a director of [the] HIPAA security, and then we have a director of the HIPAA privacy policies and procedures that need to be in place, and they govern that for the university. Now, keep in mind, which is kind of a grey area because our databases are actually on the health system network, which they have their own policies and rules, but it is supported by the university, which has their own policies and rules."

- Director of Information Services

"The IRB generally serves as a privacy board if there is any call or question raised about any of the particular issues."

- Director, Office of Regulatory Affairs

"I write all of the policies for [the] HIPAA in general. I mean... in addition to the research policies as well, but the IRB primarily does the governing of the activities with regard to [the] HIPAA. For example, I created all of the forms that we currently use for HIPAA/research that the IRB currently uses, but they are more so the police of those forms and those activities associated with the safe harbor and all of the other activities."

- University Privacy Officer

"We have a privacy officer... on things that... directly involve research – reports to the IRB, but a lot of the privacy issues have to do with operations, and so then there... that person reports to the Regulatory Affairs Office."

- IRB Director

"The HIPAA privacy officer works in the office of university counsel under the person who is the lawyer for Corporate Compliance. My institution has two distinct entities with two boards of trustees – the university and the hospital, and the hospital has their own office of legal counsel, and they have their own privacy officer but there is interaction."

-Director, Division of Human Subjects Protection

It appears however, in the institutions represented in this sample, that the health-system privacy officer typically handles disclosures of the PHI, even when the disclosure is related to research data.

Of note, in some cases we found that individuals at the same institution did not always agree about which individual or organization has the responsibility to interpret the HIPAA legislation.

In most institutions, it was either the privacy or the compliance officer with or without collaborative input who investigated a PHI disclosure. Frequently, disclosures of the PHI made in the course of university research were still investigated by the officer on the health system side (Table 14).

**Table 14:** Parties responsible for PHI disclosure tracking

Response	Count	Percentage
Privacy Officer	3	33.3

Compliance Officer	1	11.1

Either Hospital or University Compliance Officer in collaboration with IRB	2	22.2

Privacy Officer in collaboration with IRB	2	22.2

Not Applicable – Not a covered entity	1	11.1

Scenario 1 – Question 19. A total of 9 interviews provided responses. Respondents included individuals from all organizational roles. Data was aggregated with interview as the unit of analysis.

The responses suggest that policies regarding notification in the event of security incidents may need to follow very different routes, dependent on the organization. Consensus of multiple offices or organizations within the institution may be necessary. For example, it may be advantageous to ask the IRB, Office of Research, and University Compliance and Privacy Office to weigh in on who should be responsible for the local response.

### Existing identity provisioning infrastructure

Several institutions were on the verge of adopting some kind of automated, organization-wide identity management infrastructure and processes suitable for the research enterprise. Such infrastructure, sometimes called an Identity Management System, is used to construct automatic systems for creating and managing user account and access controls in many disparate computer systems within a single management domain. The process (manual or automatic) of creating and managing user identity into the systems is termed provisioning, a term we frequently use throughout this document. These institutions were interested in using this local infrastructure for eventual automated provisioning of users into caBIG users:

"As we build processes and procedures to track the people in our environment and create access for them, then revoke it in a timely fashion, it would be very easy to extend that to include caBIG and things like that. And I'd be happy to do that, and then would treat that as important as maintaining our own data, so I could step up to that obligation. But if you came to me before I had my house in order and say 'oooo... we wanna do the caBIG thing', I am not going to have the tools to really reassure you and say that we are going to take seriously our responsibility to the federation and make sure that these accounts are managed in a proper fashion."

- Information Security Officer

#### Identity provisioning and authorization of users

For many participants, the development of the caBIG federated platform could prompt a reconsideration of how decisions about access are made:

"If the systems are such that they can get into our data, we might need to think for the first time about being a little bit more circumspect and think about what qualifications we would want to impose... I think there would probably be a lot of regulatory compliance pieces we might want to spell out more than we do now."

- Legal Counsel to IRB

Many participants had difficulty conceiving of the envisioned platform and offered their insights with the caveat that additional study would be needed. Additionally, many participants had difficulty in distinguishing between authentication and authorization requirements; therefore, we have grouped these together in our analysis. Further work is needed to separate the constituent requirements more carefully.

#### Parties responsible for provisioning

Regarding the provisioning of users, there was a preference for local authority over these decisions with some caveats. In general, IRB directors were willing to consider either central or local provisioning given that data was de-identified, but were less willing to accept central provisioning if there was any risk of re-identification. However, security officers, privacy officers, and compliance officers generally preferred local provisioning (Table 15).

**Table 15:** Who should be responsible for creating identities and authorization?

Response	Count	Percentage
Local Institutions	9	60.0

Central Authority	2	13.3

Would accept either central or local	3	20.0

Depends on whether org is legal entity	1	6.7

Scenario 1 – Question 7. A total of 15 interviews provided responses. Respondents included individuals from all organizational roles. Data was aggregated with interview as the unit of analysis.

Most participants preferred to have local institutions manage the provisioning process using existing infrastructure, because they felt local institutions were best positioned to make these decisions, especially because of the centrality of the IRB to this process:

"We have to sort of credential our own people, and we know them, so...I mean I guess that with the proper credentialing checklist, a national board could do it. It just seems to be easier for local, because each local place is going to have to submit to their IRB to get a project done, or to get a project approved. So it seems to me like we would have to do it at the local level, and then doing it at a national level or more diffuse level would just be repetitive."

- Director, Division of Human Subjects Protection

"If I had to choose, I would say that each institution would create the identities or manage the identities for the people there, with the idea that the identity management ought to be closest to where the peoples' homes are. If I am going to be responsible... if I am going to have some responsibility for it, I want to be able to get out and a hold of those people, which means that there is physical proximity or an employment relationship with them, or some sort of titled relationship."

- Information Security Officer

Some participants felt that either approach would be acceptable as long as data was de-identified:

"I think we would be comfortable...anywhere in there as long as we had well-defined standards for what the authorization/certification process was. In other words... if the data is de-identified... we would be very comfortable with an external group setting the authorization and what security access to the data."

- Director, Office of Regulatory Affairs

Many participants felt that provisioning by a centralized body would simply be too cumbersome to create and maintain, and that ultimately, the responsibility belonged to the local institutions:

"Having that be fully centralized would be such an enormous undertaking, that you have to rely on certain standards and capabilities at the local site. So I really think a lot of that has to be the site becomes certified and how they can provide identities, and access, and they're audited to make sure they are doing it correctly. But I do think there needs to be some sort of central structure that oversees that."

- Director, Office of Human Research

Other participants noted that differences between local organizations could make the provisioning of users across the entire community very complex. Without a centralized legal entity, the potential for variations in the process remains:

"Well, it can be a point of weakness or a point of strength. Obviously, these people have a level of sensitivity to the individuals who are actually being granted access, and if that person does a good job and you have a very strong control measure that they did a very haphazard and poor job, then you could be granting access to a bunch of people that (a) should never have had the data or (b) that they never clean up the access when somebody leaves, so it all comes down to whether these individuals are taking their jobs seriously and doing it in earnest."

- Health System Privacy Officer

Another argument for at least some centralized provisioning was articulated by one participant who recognized the importance for having a separate credentialing body for investigators who were not affiliated with a caBIG institution. The development of Unaffiliated Investigator Agreements parallels processes that exist at the cancer centers, in which unaffiliated investigators may gain access to data after attesting to the use of a particular IRB and to be bound by the regulations of that IRB. Unaffiliated investigators would need to be credentialed by a third party.

Another participant noted that motivation to properly credential users may in fact be related to whether one's own data is "in the game". In effect, investigators being provisioned at institutions that are not providing data to caBIG may need to be treated in some ways as unaffiliated investigators because there may be little motivation to carefully adhere to the requisite policies and processes:

"One of my big motivators is that I feel a heavy responsibility to safeguard the data that we hold...If I have no data here, I couldn't care less about how the people at my institutions [handle] their identities, is set up – and that maybe that means I am a poor federated citizen."

- Information Security Officer

#### What organizational unit could credential users

Some institutions had difficulty identifying an appropriate group that could manage the provisioning process within their institution. The IT infrastructure supporting research is often meager compared with the IT infrastructure supporting clinical systems. In general, IRBs may not be well positioned to perform this task, and developing adequate control structures may be a significant task for local institutions.

"The IRBs...they just wouldn't function well in that role. I don't think there is an existing body that really could do it. We have it on one side for... our clinical data on the <system>. The business office has control. Every six months or something, they send me a report so that these people need to continue to have this level of access. On the research side, that does not exist but it could go through [the] Office of Research Administration or Office of Scientific Affairs."

- University Compliance Officer and IRB Legal Counsel

In some cases, authentication and authorization decisions require the cooperation of several groups charged with provisioning access to systems and data. This "separation of duties" is a well-accepted concept in security circles, and consequently not surprising. As a result, we should expect the involvement of a variety of local authorities at caBIG institutions.

"I think it's too new, but if I could speculate, I would say it would be a cross section between research and someone in IT security."

- IT Security Manager

#### Local governance of provisioning

Many participants suggested that it was essential to have a single individual at each institution in control of the entire provisioning process.

"We would want to create some sort of governance here, and then one local person – mediator or whoever – that monitors this on who is getting access, why have they had approval to police the access to any of the database. There would need to be an individual to govern this, I think. To me, it would need to be someone very knowledgeable of the HIPAA rules and regulations . . . to be able to police it."

- Director of Information Services

"I think you want a point person at every institution who is responsible for the various controls that are necessary for data protection... which is what I use to combine privacy and security."

- University Privacy Officer

Another important aspect of local control over provisioning was the need to have a person with authority vouch for the identity of any individual gaining access.

"I would recommend that there be one individual at each institution who is sort of top-level approver, and that top-level approver might be able to... select a next level of approver... This is the kind of thing we have done here – not in the research context – but in other access to data contexts. So you have two people basically verifying the identity of the individual, their authority to get the data, their need to know, and I think having that kind of a structure is useful. And as I alluded to earlier, you might also then have reviews from time to time of those access permissions."

- University Privacy Officer

"I think that what has to happen is that, going back to the process I described earlier is that this department chair or somebody who is privy to that individual will need to vouch for that person when they get their credentials."

- Health System Privacy Officer

#### Monitoring of credentialing process

There were a variety of responses as to the appropriate process for monitoring credentialing (Table 16).

**Table 16:** Acceptable monitoring of credentialing.

Credential Monitoring Process	Count
Periodic compliance checks with random audits	3

Annual compliance check	2

Quarterly compliance check	1

Annual Peer Review	1

No monitoring necessary if data truly de-identified and no risk	1

Scenario 1 – Question 10. A total of 8 interviews provided responses. Respondents included Information Security Officers, IRB directors and privacy and compliance officers. Data was aggregated with interview as the unit of analysis.

#### Potential for federated credentials

Very few participants were willing to answer Scenario 1, Question 8, regarding what kind of federated credentials might be acceptable. Reasons provided for the lack of response included: (1) participants had little or no experience with federated credentials, (2) it was too early to make such a decision, or (3) that such a decision would require extensive consultation with the technical security team.

#### Information needed about users to make provisioning and authorization decisions

Across all interviews, we were able to derive a set of requirements for information needed about users (Table 17). We make no attempt to define those that are required at the time of identity provisioning and those that could be deferred to authorization.

**Table 17:** Information needed about users for provisioning decisions.

Information Needed to Make Provisioning and Authorization Decisions
Institution

Federal-Wide Assurance Number of IRB

Nationwide IRB Identifier

Quality of HIPAA training verified

Has institution agreed to abide by policies?

Has institution been debarred?

Investigator

Name

Institution(s) investigator employed at

Title(s)

Is IRB Human Subjects Research Training up to date?

Is the HIPAA training up to date?***

Who has personally vouched for this individual's identity and need for access?

User has agreed to abide by policies

User has promised not to try to re-identify data

User has promised not to share credentials

User has promised to use the system only for the purposes of the project

Has the individual been debarred?

Are there findings of research misconduct associated with the individual?

Have there been OHRP sanctions?

If user associated with unaffiliated institution – has user completed an unaffiliated user agreement?

If user is performing preliminary research – has there been some other institutional review or approval?

IRB Protocol

IRB approval number

IRB approval dates

Category of IRB approval (not HSR, exempt, expedited, full-review)

PI named on IRB protocol under which user is searching

Name and short description of project

Scenario 1 – Questions 9 and 12. Respondents included individuals from all organizational roles. Data was aggregated with interview statement as the unit of analysis.

Several participants felt that the HIPAA training (although not technically required for de-identified information) would be of significant benefit if there were any chance that the information could somehow be re-identified.

An important finding from this question is the importance of establishing a relationship among the user, institution, and IRB protocol. As one participant put it:

"It comes down to you have to be assured that the person (a) has the need to access the information, (b) have (sic) gone through whatever IRB requirement, local area, local IRB imposes upon researchers in general and (c) they agree to abide by whatever agreements and standard terms or conditions that the project imposes on people who access the data."

- Health System Privacy Officer

#### Importance of verification of the IRB review during provisioning

Many existing institutional practices provide access to data derived from human subjects only after verification of the IRB review of this request:

"When it is submitted, we call the IRB approval. They submit...the search and say we have this IRB approval. They have to send us the signed IRB approval, and then we contact the IRB approval office for verification of that number – ask them the number, and they tell us who it has been submitted to and what the project is for."

- Director of Information Services

"There [are] already IRB processes in place that relate to insuring that researchers are appropriately trained, that they have the credentials – so those type of processes, I think, are valuable to go through whether this is de-identified or identifiable data, because one of the chief purposes of the IRB is obviously to control human subjects research and protect the human subjects, and there is also [a] very strong control point with regards to ensuring research integrity and the like, so I think if you abide by the IRB [a] process needs to be in place. I think you go a long way toward doing what you need to do and ensuring these individuals who are getting access to the data are going to do the right thing."

- Health System Privacy Officer

Drawing from these established practices, many participants felt that this should be captured within the envisioned system and that, in many cases, use of the system should be within the context of an approved IRB protocol:

"There should be an additional piece of information from an IRB-type committee that would say... that would at least get permission for that researcher to access the data. When the protocol is created, it can list the appropriate members and each institution will have a role configured for that person that allows him to be a part of it, and then on the opposite end, the data owner would require that role, and that kind of ties in with the governing body setting security levels and assigning risk to data."

- IT Security Manager

"From an oversight perspective...it would be nice to know when somebody [is] accessing a particular data set, that the login includes the nature of that access and whether it is preparatory or whether it is part of a research protocol, and if it is part of a research protocol, some number or some indication of what that protocol is."

- Director, Office of Regulatory Affairs- An exception to the requirement for IRB approval might be preparatory research. The definition of what constitutes preparatory research and the controls over such preparatory research appear to differ among institutions.

#### Difficulties with anonymous users

Anonymous users were considered problematic by all participants and most would simply not allow it under any circumstances (Table 18), even if the only data involved had all been de-identified.

**Table 18:** Would you allow anonymous access to data?

Anonymous Access	Count	Percentage
Would not allow	7	77.8

Would allow	0	0.0

Would allow under special circumstances	2	22.2

Scenario 3 – Questions 15 and 16. A total of 9 interviews provided responses. Respondents included information security officers, Office of Research representatives, privacy officers, and compliance officers. Data was aggregated with interview as the unit of analysis.

Many responses indicated this was simply impossible to accommodate:

"Absolutely not. There can be NO anonymity. I think that would shoot this thing in the head."

- Vice President for Strategic Planning

A few participants felt that under extremely controlled situations, this could be possible either by limiting the access technically, or by having the organizing body hold the identity in escrow.

"I think if you can establish an agreement between the private industry and the data owner that there can be some controls over how... some controls over who is accessing the data from a purely network perspective. If we can limit access to the database from a particular server, host, then that might be reasonable enough to not have user auditing. I don't think that the data should ever be opened up to anonymous access unless at a minimum something like that is in place."

- IT Security Manager

None of the participants was able to point to a specific institutional policy against this, indicating that it simply violated the norms of the institution.

#### Difficulties with accepting the HIPAA and IRB research ethics training from investigators at other institutions

Human subjects research (HSR) training is required for all investigators who work with HSR data. HIPAA training is required when data does not meet the requirements for de-identification. For current caBIG users, it is expected that users will at least need to meet the requirement for HSR training. HIPAA training may be necessary when data that may not meet strict standards for de-identification under safe-harbor is shared using the limited data set approach, requiring a Data Sharing and Confidentiality agreement. Some institutions may also require HIPAA training if data is considered to have risk other than the risk of re-identification.

An important finding of these interviews, that came up repeatedly, was that human subjects research protection training and HIPAA training may not be acceptable from one institution to another:

"And I'll tell you, our institution knows there is huge variety, and we do not accept outside institution's training."

- – Director, Office of Human Research

Respondents differed as to the best approach around this problem. Many suggested that this was an important role for the governing body to take on:

"What if our HIPAA training is much more extensive than the training – the HIPAA training at X Hospital, I would even want to know, at that [organizing body] level way up there... I would want them to also do research on everybody's HIPAA training modules to make sure that they are just as extensive and rigorous as ours is, because I want to make sure that those researchers are right on top of it and know what's expected of them, and we do not want any unauthorized access whatsoever."

- University Chief Compliance Officer

Some felt that standardization was the best option, and that such a standard could eventually replace local requirements, and would, in and of itself be an enormous step towards multi-institutional data sharing by removing a significant barrier:

"I think the best answer here is not to have this be a locally determined standard. I think there should be a national standard and a program that we could all take that would be adequate to say that we understand the basics of HIPAA, and I think there should be recertification, as well."

- Director, Office of Human Research

One IRB director suggested the Collaborative IRB Training Initiative (CITI) modules as a potential training standard for human research subject protection across the caBIG project. Similar modules might be constructed for HIPAA training specific to research uses. Individual IRBs would then need to agree to accept these modules in lieu of the local institution's training requirements.

Building on this, one participant suggested that caBIG develop its own specific training, including other kinds of training (HIPAA, research ethics, etc.) that would be acceptable to institutional IRBs and privacy officers. The advantage of this approach is that privacy and confidentiality issues could be addressed within the context of this new research paradigm. Some issues such as "fishing" which are not generally a problem in the current research paradigm could then be addressed:

"Well, you could always have online training that any potential user has to take before you give him access. [That is] unique to the grid. Especially since it is such a unique entity or beast. I think caGrid is such a huge concept that it would be a requirement that they take the training course."

- IRB Director

A potential interim approach suggested by one university privacy officer was to develop a list of the items that the HIPAA or Research Subjects training must cover, and then, upon joining caBIG, have individual institutions attest to the fact that the items in the list are addressed in the institution's existing training program:

"If you do not want to create your own HIPAA training, I would say that you want for some institutions that have done it well, you would give them a test, and in order to give them a test, you would want to say that they have [been] educated on appropriate use, educated on safeguards, educated on consequences and on resources...That might be it."

- University Privacy Officer

Individual organizations may need to retain the right to inspect the training practices of other organizations, and fairly detailed information may be necessary until some standard can be agreed upon:

"Well, first I would like to see a copy of their module and their training program. And second, I would like to [know] how often they are required to take that training....I'd want to see the actual training program itself. I want to know the qualifications of the trainer... know whether or not the individual took that training and how they scored...and how many times somebody failed."

- University Chief Compliance Officer

"Do they cover historical aspects of IRBs? Do they cover all the issues about the Belmont report? Do they cover how to consent [the] cognitively impaired? What is the yearly update? How do they do that? Do they just... rubber stamp or do they make somebody read something and get recertified?"

-Director, Division of Human Subjects Protection

#### Additional suggestions with regard to protected health information

Most respondents suggested that the situation changes greatly when data is protected under the HIPAA.

"I would expect that for de-identified it's going to be a very low standard ... I might take a blanket authorization... But when you get up into certainly the fully identified, I am going to want that information owner to sign off on each, every single request independently to say 'Yes. This is okay. This is okay.' Because that has a higher standard."

- Information Security Officer

The problem of credentialing users locally might be simplified by use of a Business Associates Agreement (BAA) that could be established with caBIG-associated institutions by the proposed non-profit entity:

"...sign the Business Associates Agreement with the medical center privacy officers of the world, which transfer, and they credential you under a different Business Associates Agreement. And they do that. I mean, they let other people get access to their PHI, if they are business associates. So, this thing develops and works with the limited data set or de-identified data demonstrated and built expertise and now [if you] wanted to get at the identifiable stuff, now you try to approach it as a BAA."

- University and IRB Legal Counsel

Another difficult issue with identified information is that passage of identified information will likely require an IRB authorization agreement. "Such agreements are not always accepted between institutions as discussed further under the topic of *patient consent*, below."

### Control over authorization decisions

#### Aspects of authorization that must be controlled locally

Although there were generally few responses to this question, the responses we did collect suggested that local institutions need to control characteristics such as roles of their own users, and that they need to control the characteristics that govern entry into their own data repositories:

"If we own the data, then we should definitely own the authorization process. We would need to have reasonable assurances that the other institution is practicing a solid policy for validating their staff. The way I see this working is what we are accepting from another institution is a validation of identity and another piece of information that says you are allowed to see the data in this particular data base, and if the owners of that data who are managing the data base can set what that piece of information is and distribute it to the participants, so if that is setting up the level for type of authorization to the data, then (that's what we need to be in control of)."

- IT Security Manager

"I would claim that I need to have control over the ability to reset their passwords or something, because they are going to come to me to ask for that. And I certainly would need to be able to control their roles that are defined in my institution, but can I envision a scenario in which Dr. Baggins is working here at (this institution) and has one role here, but he also has a joint appointment at another institution, and that's a minor thing. So his identity is verified here at the (sic) this institution BUT the other institution vouches for his role as a researcher at the other institution's protocol, based upon our identity. Yeah... that's reasonable in a federated environment."

- Information Security Manager

Most participants who answered this question were willing to allow entry into repositories that contained only de-identified data on the basis of users meeting certain pre-defined characteristics, but preferred to have a much tighter control over access to identified data.

#### Third party vs. local verification of user attributes

In general, there were few responses to the benefits or shortcomings of third party verification of user attributes. This is possibly because participants had difficulty envisioning such a remote verification of attributes. We were able to document some examples of the need for third-party verification, where the existing manual process requires some look-up of an external resource. In one example, individuals in the IRB assuring themselves that another institution has a currently certified IRB, prefer to have information about the Federal Wide Assurance (FWA) come from a source of authority such as the Office of Human Research Protections (OHRP):

"We would get it from the OHRP web site... You know... institutions are constantly letting their FWA's lapse, so we would look on the web site and ensure that their FWA is active."

- Director, Division of Human Subjects Protection

As a legal matter, at least one participant indicated that there were no specific barriers to third party verification:

"I think as a legal matter, the institution could agree to that, but the decision about what is the adequate level wouldn't be mine. It would be the IT people."

- University and IRB Legal counsel

#### Turning off access to data across the grid

Participants generally agreed that the termination of access was an important capability that they wished to retain as a right of participation in the project (Table 19).

**Table 19:** Would you want to be able to turn off data access?

Response	Count	Percentage
Yes	8	88.9

No	1	11.1

Scenario 1 – Question 10. A total of 9 interviews provided responses. Respondents included IRB directors, information security officers, Office of Research representatives, privacy officers, and compliance officers. Data was aggregated with interview as the unit of analysis.

Participants expect the ability to cease transfer of data immediately with a specific user or an entire organization, even though they felt they would use it rarely:

"If we found someone way out of compliance of what is expected, we would halt further transfer and assess whether we can continue to work."

- Director, Office of Human Research

"Well I think if I'm an institution that specifically had data, that other institutions are using, and I personally as an institution found that [there was] something they were doing wrong, I guess I would probably be inclined to take actions even before the granting authority took action. If I really found out [it was] egregious and I had to do something...I think the likelihood that it would occur would be almost never... but the value in being able to simply shut the data off knowing that you can always turn it back on versus not being able to shut it off, I think, would give the member institution some level of comfort that they have some level of control."

- Health System Privacy Officer

A number of potential reasons for termination of access were offered from participants (Table 20).

**Table 20:** Conditions where access to data should be terminated.

Potential Reasons for Turning Off Access to Data	Count
Institution non-compliant with procedures	4

Misuse of resource by investigator	3

Data found copied and unsecured	1

User accessing information not described in IRB protocol	1

Investigator disbarred	1

Access no longer needed or not being used	1

IRB Protocol expired	1

Investigator no longer employed at institution	1

Scenario 1 – Question 13. A total of 9 interviews provided responses. Respondents included IRB directors, information security officers, Office of Research representatives, privacy officers, and compliance officers. Data was aggregated with interview statement as the unit of analysis.

Participants offered a range of answers to the question of who should make the decision to turn off access (Table 21). A number of participants indicated that the data owner should be responsible for turning off access, but that some decisions regarding termination might come from the IRB or local caBIG officer.

**Table 21:** Who should be responsible for terminating access?

Who makes Decision to Turn Off Access	Count
Data Owner/Data Steward	2

IRB	3

Governing Body	1

Local Institution caBIG "Officer"	1

Scenario 1 – Question 13. A total of 7 interviews provided responses. Respondents included IRB directors and university and IRB legal counsels. Data was aggregated with interview as the unit of analysis.

#### Step-up requests

Requests to step-up the level of authorization might require additional processes, agreements, or alterations to the IRB protocol (Table 22).

**Table 22:** What may be required to authorize a step-up request?

Required for Step-up	Count
Re-verification of identity	1

Additional agreements from user regarding security	2

Amendment of IRB protocol	1

Uncertain	2

Scenario 1 – Question 14. A total of 5 interviews provided responses. Respondents included information security officers, IRB directors, and university and IRB legal counsels. Data was aggregated with interview statement as the unit of analysis.

### Auditing

Questions related to technical auditing requirements were posed to three enterprise information security experts and one hospital department information systems manager. A subset of questions was posed to selected compliance and privacy officers, and representatives of the Office of Research. Due to the small number of responses, we provide summary statements as opposed to tables.

#### Level of audit trail required

Most participants indicated that audit information was needed at data set or record level, for de-identified data. Additionally, audits needed to address logging of authorization decisions as well as access to data:

"I want to have auditing and controls, and I want to be able to say when (a person is) given authorization to use this system... who vouched for him? Who vouched for his identity to say that this is who he is. Who gave him access to this specific data? And how do I know that when (this person) ceases to have access to this data by some obscure criteria, that is going to be revoked in a timely fashion and then logged and tracked, and that motivation is purely for safeguarding the data that I have."

-Information Security Officer

In some cases, participants wanted to know who could access data at any point in time, in order to perform compliance checking on their own authorization decision-making processes, or to determine individuals who are not using the system in order to reconsider whether they continue to require access:

"Definitely, ahead of time, we would want to know which researchers have been approved – pre approved – as the bona fide staff or faculty that may have access to that information."

- University Chief Compliance Officer

"Well... I would want to know that institution B is reviewing every year, who has access to this system, whether they still need it, and whether they still need that level of access... If someone has not used it in six months, chances are they really do not have a need to know. "

-University Privacy Officer

#### Generation and Management of Audit Data

There was a consensus among the participants representing information security that local institutions should generate the audit data and that some central authority should aggregate, analyze, and distribute aggregated and analyzed data back to the local institutions. Participants wanted to retain the ability of the local institutions to inspect all relevant auditing data, in order to evaluate the sufficiency of any central investigation process, and also because in some cases they must conduct their own investigation because they are the responsible party:

"If it doesn't have the ring of truth to it, I am going to want to go out and do my own investigation to verify... Even if it does have a ring of truth to it, I may have my staff do some cursory investigation. Do a little bit of fact checking on this....just to make sure... and... I would like to have the tools and the ability to do that."

- Information Security Officer

"There has [to] be an understanding that each institution is allowed to review the others' process both in terms of what their process is and some sort of follow-up audit process to ensure that."

- Institution Compliance Officer

"...but I may further want to be able to say that as an information provider, who has made data available to this consortium – this federation . . . that I need the ability to go in and audit the auditors or go in and look directly at the people. . . the audit trails for the people that touched my data to reassure myself that this is really working, because it's . . I want the centralized community to do the heavy lifting. I don't want to really be doing all this scrutiny and stuff, but it's still good for me to periodically make sure that things are working as proper . . as they should . . so I think I would like to have the ability to peek in now and then, as well."

- Information Security Officer

Participating institutions need to feel that they can trust any centralized security incident management processes:

"Where it would become awkward is if an event occurred, the centralized body gave me a report, and it was crap. If it didn't have the detail – if it didn't have the data, if it looked like somebody was throwing squid ink at it . . . at that point, the whole relationship is in jeopardy."

- Information Security Officer

#### Time interval for maintaining access log

Time intervals suggested by the four participants varied markedly. Responses included: 45 days, 60–90 days, 6 years, and forever.

For de-identified data, there are no specific guidelines; however, other requirements that were suggested in these interviews may end up being the determining factors in planning retention times. For example, one potential factor in establishing the appropriate audit log retention time, is dealing with issues such as scientific misconduct:

"I think [another issue is having a] scientific misconduct policy...say you have an instance where from some IRB perspective, you have... non-compliance, I think there should be a provision that those instances are disclosed somehow, because you don't want to have data in there that was not consented properly. That ruins everything. Let's say you have a physician that had enrolled patients in the trial and had shared data, and maybe it was de-identified and went through all the correct procedures. After the fact, we find out that 40 of the 50 patients were not consented according to the approved IRB protocol. So, by scientific misconduct standards, that data [should no] longer be available for... research."

- Director, Office of Research Administration

If we need to be able to contact all investigators that have used such potentially tainted data, we may need to preserve audit logs for a much longer interval. This could be true even for de-identified data. It should be noted that it will likely be necessary to "quarantine" the affected data – that is, turn off access to a broad group of people, potentially everyone using the system – and contain the affected data indefinitely during an investigation and remediation period.

#### Effect of level of identification

Interestingly, although many participants felt that other controls would differ markedly among the different types of data (de-identified, limited data set, and identified), the same individuals generally felt that auditing requirements could be, and should be, essentially identical regardless of the type of data:

"My audit requirements are the same for all of them. I want to know who is accessing stuff regardless."

- Information Security Officer

Unlike processes that could pose barriers to research, audit data is only a technical burden; therefore, the benefits of having access to this information may outweigh the costs:

"I would also say too that even if we are dealing with de-identified data, you'd want to take a conservative approach and still, set the standard of what a limited data set requires in order to provide those conditional protections just in case. This is a good argument, especially when we are doing a multi-institutional type of study that you might want to employ."

- Health System Privacy Officer

#### Impact of workflow tools on Auditing Requirements

Participants voiced concerns about workflow tools and other processes that result in derived data. Here we use the term workflow tools as a generic term to refer to mechanisms that allow a series of operations and the data flows between them to be modeled, and carried out in an automated fashion. A well-known example of a tool for scientific workflows is the Taverna [29] workflow system. This question is of relevance as caBIG™ is developing this capability as part of the caGrid tool suite. In particular, the director of information services we spoke to who directly controls a clinical database was very sensitive to the idea that these class of tools might alter the initial data and falsely represent it to the user:

"Another thing that will concern me too, is how do we know the integrity of the data has not been altered.... I would want to... verify that the data is still the same."

- Director of Information Services

The passage of identified data through third party workflow and analytic tools posed a particular concern, and greatly increased the requirements for auditing:

"If you are passing fully identified data sets, these third parties have to have agreements in place – full confidentiality, security... You need to be qualified vendors in the sense that someone who is assessing them they have the policies of the institution correct – they are implementing them and periodically are audited. That would be high-level scrutiny for them. And then, it's just a matter of having some typical thing you'd want from a vendor anyway about the reliability of their processes. And from a software standpoint, you know very well there are all sorts of validation checks that need to be done, and you would simply just look at their validation SOPs and it would be up to each user to decide whether they wanted to do additional validation to prove their validation. So I think once you've solved the security issues, the rest is more like a traditional effort you might do with any vendor, looking at validating their IT processes."

-Director, Office of Human Research

#### Auditing data portability and secondary uses

Several participants raised concerns about the portability of data (from an IT perspective) and unauthorized secondary uses of data (from an IRB perspective), and cited the need to audit data portability as part of our standard compliance checking. Appropriate measures would need to be developed to deal with such unauthorized secondary uses of data:

"I'd like to see security policies around the portability of the data. You know, a lot of people work on laptops now. It's more common to see laptop users than desktop users. That brings out a whole new security issue. That's probably the bigger issue – this is so much... that here, you've got the data and how the thing is stored and collected, etc., we now come to somebody's laptop... and how that's being managed."

- Director, Office of Human Research

" [We would to turn off access] if we found data laying around – that would really be easier for me to determine from here – if we found hard copies of data laying around or something like that, we would strip them of that."

- Director of Information Services

"I think the issue that we have here locally is continued access to data sets that are pulled out of something like caBIG and secondary uses of that data, so we think that if somebody is creating a secondary use, that we do not have very good policies. I am not sure any other IRB does as well, as to making sure that an investigator has particular data, understands that he has it for one use, and at the end of that use, it be destroyed or a separate application to maintain it has to be developed. If de-identified... the risk is very low . . .but if you move up to containing identified or re-identifiable information, then the use security risks of having multiple little data sets sitting around on computers that have been downloaded from a central repository becomes fairly significant."

- Director, Office of Regulatory Affairs

#### Auditing compliance with IRB protocols

IRB directors and Office of Research representatives were particularly concerned about the potential for inadvertent misuse of the caBIG. In particular, the enormous volume and variety of data available could increase the chances of "fishing expeditions", where investigators hunt through data without a specific purpose in mind.

"So if I requested I wanted to go in and look at... whatever data is in there...6-year-olds having X disease in the year 2000, I shouldn't be able to get everything else that is in there."

- Institutional Compliance Officer

"I think that what worries people about this project is that you could have ...50 users identified at each site...sort of willy-nilly go into databases and pull out stuff and then decide about a project or start analyzing data."

- IRB Director

There are two potential approaches to the problem of "fishing". Both approaches require that the investigator present their IRB credentials at the time that they are provisioned. Users can be required to attest to the fact that they are using data for a purpose sanctioned by their IRB.

"I think that – I know that the people here would be much more comfortable if they were assured that users were investigators who had a legitimate purpose for wanting to use the data... I mean actually, what we would like is to say – all right, this person has a given project that has been approved and that the PI for that project – the user for that project, and those he designates to work on the project – give assurances they are going to use the data only for that project."

- IRB Director

Attestations might be made in the Authorized User Agreement, but could also be reinforced by reminding users each time they log into the system.

A second possible approach that arose from the interviews is that we could audit users to be certain that they are in fact viewing data that relates to their identified purpose.

"In the terms of whatever in signing on to use the grid, that the expectation is that they will be randomly checked, and if any uses are outside of IRB approval or lack of acknowledgement of the resource, they are out."

- IRB Director

The need to audit compliance with an IRB protocol further supports the need to capture sufficient information about the approved protocol to judge the intent of the research. It also suggests that data record may be the minimal level of auditing required even for de-identified information.

### Response to security incidents

#### Information Needed by Local Institutions in the Event of a Security Breach

We aggregated all information that would be requested in the event of a security breach in Table 23.

**Table 23:** Information needed in the event of a security breach.

Information Needed in the Event of a Security Breach
Investigator

Name(s) of individual(s) responsible

Who funded the project?

Description of the project for which the data was accessed?

Data Accessed

Description of data accessed

Risk level of data

How many patients/participants/subjects were affected?

Were any identifiers present in the data?

Was any data modified – Is the integrity of the data still intact?

Dates of access

What period of time did the data cover?

Incident

Was data re-identified?

Where (physically) did the breach take place?

How many times was the data accessed?

Was the data accessed by more than one individual?

Was data made publicly available (for example on a public website)?

What state did the security breach occur in?

Were SSNs or other financial information released?

Management

What discipline was provided at home institution?

Who was responsible for maintaining security of data?

How was the incident discovered?

Who discovered the incident?

What was the chain of reporting once the incident was discovered?

Was there a failure on the part of the local institution?

What oversight did caBIG governance have over matter?

Was there an unaffiliated investigator agreement in place?

Scenario 3 – Question 17. Respondents included individuals from all organizational roles. Data was aggregated with interview statement as the unit of analysis.

#### Reporting requirements

Reporting requirements will vary depending on the incident, type of IRB approval, and state where data was collected. Examples of entities that will require notification in some cases include: (1) IRB of the providing institution, (2) IRB of the receiving institution, (3) funding agency supporting the research, (4) OHRP, and (5) patients in NJ and PA (if financial or SSN disclosed):

"Certainly, there is reportability back to the IRB. There is probably reportability back to our audit committee of our board, which oversees compliance in HIPAA at the very minimum, depending if it's identified... I mean, it could go all the way out to notification... certainly notification to the patient if it's identified, but also perhaps the government agencies."

- Institution Compliance Officer

"And if they were doing something that was against their own IRB's approval, they would have to be reported to OHRP or Research Integrity."

-IRB Director

"If any federal funds were involved in the research that use [s] the grid as a source, they would report it to OHRP. They would report it to the funding agency."

- IRB Director

### IRB issues

An important finding of this research was that, in general, participants accepted the idea of a two-protocol mode for data exchange. In this model, both the repository owner and the investigator (who may be at different institutions) may have IRB protocols from their respective institutions. This is true as long as all understand and agree to this approach upfront:

"As long as the institution or group or investigator realizes he is putting information samples, whatever, into a repository that is going to be available to other investigators, and they are going to be reviewed by their board... so that's fine."

- IRB Director

#### Protocol required for setting up a de-identified repository

There was marked variation in the class of protocol that institutions were likely to require to establish a data repository for caTIES, ranging from no HSR waiver to expedited review (Table 24).

**Table 24:** IRB protocol required for establishing repository.

Response	Count
Not Human Subjects Research determination	1

Not Human Subjects Research determination OR Exempt	1

Exempt	1

Exempt OR Expedited	1

Expedited	1

Scenario 2 – Question 1. A total of 5 interviews provided responses, from 5 institutions. Respondents were IRB directors. Data was aggregated with institution as the unit of analysis.

#### Protocol and agreements for searching a de-identified repository

We did not specifically ask about the kind of protocol expected for the investigator at the receiving institution, but several IRB directors offered that the protocol would likely fall under a "Not Human Subjects Research" or "Exempt" designation if only data was exchanged. For tissue exchange, there was a wider variety of responses.

For data that is potentially re-identifiable, existing IRB processes generally also utilize a Data User and Confidentiality Agreement between the data provider and the user:

"We have other repositories ... research data. These have been de-identified or it's data that is still identified but is collected under an informed consent that allows a person secondary research. And when we have those kinds of databases, we allow people from other institutions to access. They access them pursuant to a data use and confidentiality agreement to limit the use to whatever the approved research is and to agree not to further post, redistribute etc. And then we would require that they provide their IRB approval for what their research is. . . It may be exempted . . expedited . .dependent on what it is they are doing and what kind of data they are getting. But for right now, we would consider that our responsibility to ensure when that data goes out there, it is under an appropriate agreement limiting the use by that person to something that is either approved human subject research or not considered human subject research under the federal [guidelines]"

-University and IRB Legal Counsel

As articulated by one privacy officer, a potential problem with Data Use and Confidentiality Agreements in relation to use within a federated data-sharing environment is that they are typically specific to a project and executed between the researchers and the other institution:

"I'd have to go through the [regulation] but there has to be some relationship then back to the researchers just not the institutions. Again, I think . . . you can't assume that it's institution to institution. It's institution to researcher who happen [s] to be affiliated with another institution."

- Health System Privacy Officer

#### The Problem of data outside the scope of consent

An important problem we detected with the current assumptions regarding IRB protocols for grid use is that data to be obtained under many protocols is bound by the dates with respect to the IRB protocol. For exempt studies, data obtained must have been collected prior to the granting of approval. This constraint is not required in cases when the research has been designated "Not HSR".

"For exempt – it exists. It's on the shelf. There is no intended forward stuff going on, and then if it is, it's minimal risk and you do a waiver, because... you [need to be] assured of its safeguards. But then if it's just not human subjects research, all that goes right out the window."

- *IRB Director*

It appears that this requirement is true only for the investigator-initiated IRB protocol, and not for the development of the repository. Assuming data will flow continuously into caBIG repositories from other sources, it may be necessary for the grid system to regulate the release of data in accordance with this constraint. In order to provide data to a particular user, the system must know the IRB type, and the date of approval of the IRB protocol.

#### Use of aggregate data

Most IRBs felt that use of aggregate data (for example as histograms) would not be considered human subjects research, and would, therefore, be suitable for preparatory research (Table 25). However, at least one IRB director felt that data needed to be physically separate and not simply an aggregate view of more complete data sources.

**Table 25:** Is aggregated data considered to be Human Subjects Research?

Response	Count	Percentage
Yes	1	20

No	4	80

Scenario 2 – Question 18. A total of 5 interviews provided responses, from 5 institutions. Respondents were IRB directors. Data was aggregated with institution as the unit of analysis.

"You would have to go to a separate repository to do that because it could . . . you could not give access to the public for that within that. . . You would have to take the data out of the caBIG system that he needs and import it into a separate secure system that would be public access. But if you want to give them access to the data so that they can manipulate it themselves ... I mean, you have expanded your audience to potential people who have access to data which they could attempt to re-identify without additional security that would be built into the caBIG access. So, you're providing to the network some assurance that Joe Blow at some other institution isn't going to do that and allow secondary access. It would be (sic) really be consider [ed] secondary access, which would increase the probability that somebody potentially could re-identify that data to a local level."

- Director, Office of Regulatory Affairs

#### Importance of defining a level of risk for IRB approval

The importance of risk level for making authorization decisions has previously been discussed. Assurance regarding the risk level at the providing institution is also important for securing IRB protocols at the receiving institution. Thus, an aspect of the approval process for caBIG repositories that needs to be addressed through agreements and/or auditing is the assurance that information in the repository meets the definitions of the appropriate risk level – for example de-identified data under the HIPAA safe harbor. Individual IRBs must have this assurance in order to approve the protocol on the investigator side:

"The other IRB would have to be assured . . .and know that the data that the person was getting is in fact de-identified, which it would be in the repository."

- Director, Division of Human Subjects Protection

For this reason, local caBIG repository owners and stewards need to be able to define and attest to the risk level specific to their context and state law. Sharing of data must operate under these constraints.

### De-identification

Assessing the risk of imperfect de-identification is an expected local IRB function evaluating an IRB protocol to establish a caBIG repository such as caTIES.

Not all institutions limit their definition to that which is provided under HIPAA (Table 26). Additionally, it appears that in some cases state law may supersede the HIPAA definition of de-identified, further complicating the matter of establishing uniform policies across a federated grid:

**Table 26:** Does your institution have a more specific definition of de-identification than the HIPAA?

Response	Count	Percentage
Yes	1	20.0

No	4	80.0

Scenario 2 – Question 12) A total of 5 interviews provided responses, from 5 institutions. Respondents included individuals from all organizational roles. Data was aggregated with institution as the unit of analysis.

"The thing... I am worried about is because your are setting this up in such a way that you are in fact creating a highway for data... the rules of which each supplier (of) data has to comply with are going to differ, and... that includes whether or not something is de-identified. So in Washington State, for instance, the state law considers DNA information as individually identifiable information, so even if you took out all of the identifiers, that HIPAA dictates be removed in order for it to be de-identified... So health care information is considered identifiable health care information under Washington law if it contains DNA information.... You could never de-identify it under Washington State law."

- Legal Counsel to IRB

In the case of Washington State, some have suggested that state law may be interpreted to forbid transmission of sequences from patient material, or even prohibit the sharing of tissue from which DNA might be extracted.

The responsibility for assessing the adequacy of de-identification for patient related data appears to rest very clearly with the health system or hospital. However, the use of an honest broker to act as an intermediary between the identified clinical side and the de-identified research side benefits both sides. The honest broker can thus take on some roles of a data steward in assuring that data in a particular system does not exceed the level of risk that later IRB determinations are based upon:

"Now, when you say if there is one date, are you saying that by accident it occurred? To me, that is a whole different issue. I mean I think that whenever we talk about de-identification, there is always the potential that somebody screws up and something gets in that should not be in, and frankly, that does happen under our IRB rules. That is considered an unexpected event presenting a potential risk to the subject and would be required to be reported to the chair of the IRB who would then consider whether ...further action needed to be taken. [With regard to the determination that the data is de-identified]...to me, that is a medical system issue... what they think is an adequate system to be identified, recognizing the risk that things happen. So from my perspective, that's the hospital's decision about what is adequate for PHI. The way the IRB has it set up, the medical system has to certify the honest broker. If they certify the honest broker, we accept their determination of what is adequate."

- University and IRB Legal Counsel

#### Reducing risk of partial de-identification

Respondents were asked how they would reduce the potential for incomplete de-identification if automated processes are employed, as envisioned in the caBIG project. Automated de-identification of free text has a number of challenges, including recognition and preservation of contextual information. For example, although proper names in a text document must be removed, the subject of an action in the text (i.e., Physician, Nurse, Patient), must be preserved. Consequently de-identification algorithms occasionally leave information in a document that allows a human reader to infer identifying information. The risk of this information varies from full disclosure, as in the case of a proper name, social security number, or other identifiers, to limited; as in the case of missing the removal of a birth date or other personal attribute (Table 27).

**Table 27:** Additional measures suggested if de-identification may be incomplete.

Additional Measures for Potentially Incomplete De-identification
Quantify risk prior to establishing repository with biostatistics consultation

Provide test data for human review

Include QA mechanisms in Data Safety Monitoring Plan

Perform periodic random checks to assess completeness

Scenario 2 – Question 7. A total of 3 interviews provided responses. Respondents were IRB directors. Data was aggregated with interview statement as the unit of analysis.

"If I know that there are really, really technical controls to factor authentication, only one machine always patched, firewalls, strong authentication, regular review, it makes me a lot less worried about the occasional re-identification. There's not a magic bullet for privacy or security. It has to be a whole combination of things... do your gosh-darn best to de-identify, and whatever you can't get to, depending on your comfort there... you have to step up more controls if you feel like you are really just not getting to a level where you can be sufficiently comfortable."

- University Privacy Officer

#### Risks that go beyond accidental or intentional re-identification

Although de-identified data does minimize some risks, many respondents were quick to note that even truly de-identified data did not mean risk-free data:

"The reality is that even if it's de-identified data, I still have some measure of responsibility over the data that my institution provides, and so there has to be some understanding that the researcher...that the data is still some institution's data, and it is a privilege for them to have access to it."

- Health System Privacy Officer

"The fact that it's de-identified and therefore qualified as not human subjects research, that would get the IRB out of it, but that's not going to necessarily get the institutional concerns out of it."

- University and IRB Legal counsel

### Patient consent

#### Acceptance of consent forms from other institutions

Four of the five IRBs indicated that they would generally accept the consent form of another IRB. However, it seemed unclear whether the case-by-case decisions that governed such acceptance would really scale to caBIG. Some participants saw acceptance of consent forms from other IRBs in this federated environment as especially problematic. One IRB director advised that a common consent form agreed to by the participating IRBs was the only way to avoid the need for point-to-point decision-making by individual IRBs:

"It's something that has to come from this governing agency; a common language, and then the individual IRBs will have been involved in that, so that they are all or mostly all on board. Because what you don't want to have happen is... you don't want the individual IRB language, because once they mess with the language, there is inconsistency. You won't be able to use this across the different sites...And I think that this is the biggest deal killer I can imagine, quite frankly, because the IRBs will not agree with one another. They are very disagreeable."

- IRB Director

Developing a common consent form would enable multi-institutional prospective research projects, but would require strong NCI leadership and involvement of the individual IRBs, including face-to-face meetings of IRB representatives to agree on a common form:

"You have to have that leadership, and the people who do this, have to be practical. They have to really understand these issues and the complexity of these issues. And I think there does have to be some kind of consensus. This is a situation where it would be useful for NCI to have a couple of focused consensus meetings where they deal with this... and perhaps periodic telephone or video conferences, because if this has to go full board, for example, somebody from this IRB has to be in the committee meeting to justify why we are doing it this way, which may not be the way we would ordinarily do certain things. Part of this educational process – every single IRB is different. That's the strength of the IRB system, and the weakness of the IRB system. There has to be a buy-in. There has to be real ownership. And I think that what IRBs get out of this is they learn more about how to think about the consent process. And frankly, I think all the IRBs around the country – most of the IRBs and a lot of the small ones are very interested in these issues. We understand that the process does not feel right, right now. So I think that's what incentives (sic) the IRB to participate in this."

- IRB Director

#### Elements needed in the consent form

All four IRBs indicated that development of the repository must be indicated on the consent form (Table 28).

**Table 28:** Must the development of a repository be indicated on the consent form?

Response	Count	Percentage
Yes	4	100

No	0	0

Scenario 4 – Question 6. A total of 4 interviews provided responses from 4 institutions. Respondents were IRB directors. Data was aggregated with institution as the unit of analysis.

If IRBs are willing to accept a separate consent form, this may pave the way for a caBIG-wide form with common language that could be appended to studies generating caBIG bound data.

"It should probably be separate from the study consent... but it should [be] an NCI sponsored, cancer initiative consent that says 'Our institution participates in this initiative. It is designed to create a highway of information and tissue that will hopefully expand the speed at which cancer research is done', and explain it in fairly great detail to the extent that you can... and give them all the bad news... the bad news being that information about you will be used by researchers all over the world. Your tissue will be put into a repository. It will be manipulated . . . find out if they say yes."

- Legal Counsel to IRB

#### Re-consenting of human research subjects

One participant noted that a significant security breach might have the effect of requiring re-consent of patients because the risks of participation would be altered (Table 29).

**Table 29:** Situations where reconsenting of research subjects may be necessary.

Situations Where Subject Reconsent May be Necessary
More specific purpose than indicated on original consent form

Secondary testing creates data with new risks

Genetics Testing

HIV testing

Security of original data compromised

Patient/Subject has turned 18

Scenario 4 – Question 8. A total of 6 interviews provided responses. Respondents were IRB directors, university and IRB legal counsel, and an Office of Research representative. Data was aggregated with interview statement as the unit of analysis.

"We have lots of reasons for re-consenting or reauthorization, depending on whether or not we believe the risks of their participation change, so if there is a major problem with a security breach or something, we may require the investigators [to] go back and at least make an attempt to re-consent or reauthorize the use of a particular data set."

- Director, Office of Regulatory Affairs

#### Waivers of consent as an alternative to re-consenting

An alternative to re-consenting in some cases may be to obtain a waiver of consent. As one participant pointed out, many important existing databases were obtained without explicit consent for sharing of data, principally because technology for such sharing was not yet envisioned. Further, the keys that would allow re-consenting have been destroyed according to the original protocols:

"At the time that many of these huge databases that currently exist or were created, there was never any expectation necessarily that the technology would reach a point where data sharing of the type you are trying to design would take place. So, people were promised that any information about you will be kept confidential. It would be only be shared with those on the study staff, and any use of it will not have any identifiers about you unless it has been approved by an institutional review board in accordance with law, and when we are done with this study, we will destroy the data."

- Legal Counsel to IRB

Waivers of authorization are a problem, because individual IRBs may not accept each other's waivers:

"Frankly, I don't think individual institutions are will [ing] to accept other IRB's waivers and authorizations."

- Health System Privacy Officer

Another participant suggested that it would be very advantageous to have uniform language regarding security safeguards that could be used by local institutions when applying for a waiver of authorization from their IRB.

#### The problem of undefined future research

Undefined future research is a significant problem with prospective studies that IRBs approach differently. Some IRB directors we spoke to indicated that they encouraged investigators to use the broadest possible language still acceptable to the IRB. Others preferred to let protocols remain rather specific to discourage undefined future research. Two participants noted that the frequent changes in consent form language could be a significant impediment to using the grid for as yet undefined research and that it was therefore critical to deal with the consent issue as a community.

In the case of identifiable data, the problem of undefined future research is made even more complex by the privacy regulations. As one participant notes:

"The fact is that HIPAA seems to require a sort of a project-specific authorization."

- Legal Counsel to IRB

One respondent considered the provision for undefined future research to be especially problematic given the multi-institutional nature of this project and the existing IRB processes for handling waivers based on adequacy of security measures:

"We do allow there to be a research protocol that allows people to be entered into registry for future research, but HIPAA does not allow you to collect data for future undefined purposes. So what we are doing... normally, how it would go is that the authorization will allow us to collect the data, then further authorization would need to be used to research that data in a different way or waiver of authorization from the IRB assuming that they have sort of verified all the security measures are in place...We put an indefinite kind of time period on it because we are allowed to do that, but we also make clear to them in the authorization that we cannot do anything with that data unless we get their authorization or have a waiver. That gets into my issue with the huge gap on a national level. We use our IRB to determine whether there are adequate security measures in place to waive the authorization and use the data for another purpose. If you aggregate the data, you would need some sort of national entity that would do that."

- Director, Office of Human Research

## Discussion

Building effective security systems for a project of the size and scope of caBIG remains a complex and challenging, although manageable, task. The legal and regulatory landscape is difficult and evolving, with the current rules and regulations being interpreted inconsistently by various institutional review boards and regulatory bodies. The grid concept, and indeed the concept of caBIG, is predicated on the ability to share data freely in a federated fashion. This implies supporting technology, supporting business and legal agreements between parties. The current practice of using various point-to-point agreements to facilitate data sharing will not scale to the size envisioned. Reducing the complexity of a system from one that grows as the square of the number of interconnections to one that is linear in the number of connections is a well-known and well-accepted principle of systems theory. Here, the system we speak of is not technical machinery, but rather the set of documents, agreements, policies and processes required to create and sustain an effective federation. Realizing this system is as much an exercise in social engineering as in software engineering.

Below we discuss the important issues and high-level recommendations resulting from this work (Table 30). Where the authors believe an important project assumption has been verified by the interviews, the conclusion stands by itself with no further action suggested. In most cases, additional and ongoing effort – consisting of further study, consensus building, and organization leadership – will be required. In some cases, there are clear steps that appear to be possible to build supporting structures, both of governance and of infrastructure that would greatly facilitate approval of federated systems by local IRBs and other institutional officials charged with compliance oversight.

**Table 30:** Summary of security and privacy requirements for a federated biomedical grid.

Guidelines
A separate legal entity for governance is desired.

Consensus on foreign and commercial partnerships should be developed

Risk models and risk management processes for data within the Federation should be defined.

Specific technical infrastructure to support the credentialing process in the regulated environment should be developed.

The feasibility of creating a federated honest broker system should be studied.

Local control of identity provisioning and authorization of users is desired.

The identity credentialing process should be strong.

A special credentialing structure for institutionally unaffiliated investigators will be needed.

Existing institutional infrastructure should be leveraged.

Develop or acquire acceptable HIPAA and research ethics training modules for the entire federated community.

A central auditing authority is a necessity.

All data sets dealing with human data, whether de-identified, limited, or fully identified, should be subject to the same auditing requirements.

Specific tooling to support the auditing functions is needed.

A Two-protocol Mode for Data Exchange is accepted by interview participants.



**Further Study**

Potential for federated human honest broker systems to reduce the number of cases where identifiable information is necessary.

Manner in which undefined prospective research involving data and tissue repositories will be consented and handled.

Establishment of data use and confidentiality agreements between participant organizations and individual investigators in a scalable fashion.

Development of common consent forms acceptable to all IRBs participating in a federation.

### Construct a separate legal entity for governance

The major recurrent theme that emerged throughout the interviews, either directly or as a logical consequence of verbal statements made by the interviewee was the need for a clear, cohesive, and empowered governing entity. This is similar to the conclusions reached by the European Advanced Clinical Genomic Trials on Cancer (ACGT) project, which concluded that a separate data management board was needed [30]. Interviewees stated that a governing body was a necessity for effective operation in areas where exchange of regulated data (de-identified or identifiable data) was taking place. It was suggested that this body must be a separate operating entity, possibly a non-profit entity. The governing body must have oversight and accountability to the user community in a variety of areas. These responsibilities include accreditation of participating entities, policy and enforcement authority in the areas of data use, risk assessment, security policy and procedure, auditing, compliance, dispute resolution, indemnification, and liability allocation, among others (for a full description, see Table 5). While small scale pilot operations can be built and sustained initially with a small number of institutions, large scale federated efforts must include a legally separate governance structure for areas involving regulated information exchange. This will have direct bearing on various business arrangements between participants, security policy, and potentially the technical implementations of the underlying security system. Failure to recognize these areas and take supporting action will likely limit usability, slow the broad adoption of key components, and ultimately threaten the sustainability of data sharing federations. An important incentive to develop such a governing body is the indication that with sufficient governance structure, point-to-point agreements would not be obligatory. New organizations could join the federation if they agreed to adhere to master document sets and agreed to audit to demonstrate compliance with the same.

It also appears a key task of such a structure will be to provide a mechanism to support trust brokering among institutions that have different quantities of data exposed, or among those of substantially different size and sophistication. Without this mechanism, some large data providers may have reservations about releasing even de-identified data unless mechanisms exist to reliably verify compliance to a minimum set of standards by the consumer of data.

### Develop consensus on foreign and commercial partnerships

Regulatory groups have serious concerns about data sharing projects aimed at including foreign and commercial partners. At least some of these concerns stem from the perception that foreign partners may have higher, not lower, privacy standards.

Similarly, data sharing with commercial entities is viewed as a problematic issue, but for reasons involving improper use of data. An interesting topic that emerged from these interviews is the issue of private inurement – specifically, can non-profit participants provide data free of charge via a federated system without receiving value in return from the commercial partner? Once again, establishing a governing membership body funded by membership fees would probably limit the impact of this issue entirely because derived commercial value could subsidize operations costs and therefore reduce membership fees to the non-profit members.

### Risk models and risk management processes for data within the Federation should be defined

Appropriate decision-making on security and privacy issues derives directly from the characteristics of data and the processes involved in the handling of data. In addition to being verified by these interviews, this constitutes well-codified security principles spelled out in standards documents such as ISO 17799:2005 [31]. An appropriate risk model should take into effect state and local law, and contextual issues as well as more global aspects such as IP value, clinical vs. de-identified vs. exempt/non-human data, and re-identification risk. At a minimum, such models should include the risks to data, repositories, and institutions. Those dealing with de-identified data must include some assessment of the likelihood of re-identification. Existing, best practice frameworks for IT governance describe risk management methodologies in detail. At a minimum, the standards indicated in Federal Information Processing Standards (FIPS) 199[32] should be used to categorize the elements of the risk model. Indeed, depending on the precise governance model selected, if elements of a federation are ultimately classified as part of the Federal Information Security Management Act of 2002 (FISMA) [33], this may be a legal requirement. Those seeking to develop large-scale data sharing federations should not try to develop their own method ad hoc, but rely on established and mature IT and risk assessment literature and practices such as the CobiT 4.0 framework [34].

### Develop specific technical infrastructure to support the credentialing process in the regulated environment

A specific area identified during the interviews, which would facilitate data sharing, is an online registry of "accredited" participating signing organizations. The concept of having an online support infrastructure for protocols, trust and security levels, IRB federal certification, and other metadata to support the regulatory process decision-making process emerged in several interviews. It is a requirement that regulatory and compliance personnel be able to determine – possibly ahead of time – who can access what data under what circumstances.

### Study the feasibility of creating a federated honest broker system

The interview process suggested that honest broker systems are of interest to the community to enhance data sharing. Importantly for a federation, structured use of such systems could reduce number of cases where it is necessary for identifiable information to leave local control.

From a systems architecture perspective, honest broker systems can be thought of as a design pattern containing a requestor and a publisher. Consequently, data sharing projects that develop software would be well served to consider this a high level architectural model for constructing software.

### Identity provisioning and authorization of users

The interviews surfaced substantial information on requirements of identity provisioning and authorization of access to data. Key points and recommendations are presented below.

Our data suggest that local control of identity authentication and authorization issues is preferred by the majority, although a significant proportion of the respondents believed that central identity provisioning was a viable possibility. Most of the participants in the interviews had trouble understanding the concepts and implications involved with a federated environment, even though federation of identity and data sharing practices represent a model that retains and enhances local control. Further work on user education and actual experience will be needed for groups to achieve comfort with the concept of federation. Respondents felt that responsibility and accountability require local control in such a system; however, it was noted that because of the differences in practice between institutions, a centralized legal entity is required to coordinate and enforce policy and practices.

#### Create a strong identity credentialing process

This study highlights significant concern about the strength and robustness of the user credentialing process (identity vetting) available within local institutions. A number of reasons were given for this, including institution size, the amount of data a given institution serves to the grid, differential financial support for clinical computing and research related functions, and organizational structure and mission of the groups performing the credentialing process. There was a feeling that even though IRBs need strong auditing and credentialing safeguards, they may not be well positioned or staffed to actually perform credentialing functions. There was also strong desire to have a single identified individual at each institution be accountable for and in control of the entire credentialing process. This includes having processes in place to verify identity of users (what we would term "providing authentication functions"), and to perform authorization functions such as the association of a person with particular research roles and allowing access to information restricted by specific IRB approved protocols.

Local control of both authentication and authorization can be facilitated by the use of a common certification authority (such as Verisign™ [35] or SAFE-BioPharma™), using a common certificate policy framework consisting of a certificate practice statement and certificate policy, and a registration agent certification process available to each institution participating. Designated staff at each institution could be certified as registration agents (RAs) by the managing body of the certificate authority. The registration agents would then issue credentials to end-users. This use of common practices and certification creates a common and uniform chain of trust between all parties involved in the federation. Development of such practice frameworks should alleviate concerns expressed by IRB members about institutions with insufficient internal policies. Such frameworks are used in a number of successful federation efforts, such as SAFE™ [26], the University of Texas Health Science System, and the federal E-Authentication [36] effort.

#### Create a special credentialing structure for institutionally unaffiliated investigators

In a federation where the basic membership level is expressed at the institutional level, it may be beneficial to develop mechanisms that allow individual investigators who are not affiliated with member institutions to access or even contribute data to a federation in a secure fashion. As with foreign and commercial entities, unaffiliated investigators pose significant challenges to the establishment of a trusted federation. Most notably, they are not employed by a participating entity and therefore may have less incentive to avoid breaching their agreement to participate. Consequently, they may require more monitoring and control by the centralized governing body. Federations must develop mechanisms to deal with this issue.

#### Take steps to leverage existing institutional infrastructure

Several institutions interviewed have or are on the verge of adopting centralized identity management systems. Not surprisingly, participants expressed a desire to leverage this infrastructure for any federations that they may join. This parallels the situation among InCommon members where institutional identity management and a central university authentication authority is used for all systems within a security domain. Indeed, many universities require all information systems to use these institutional identity services for authentication control. Developers of data-sharing federations should consider the preferential use of centralized identity management systems.

### Develop or acquire acceptable HIPAA and research ethics training modules for the entire federated community

The interviews revealed clear difficulties with the acceptance of external HIPAA and IRB Research Ethics training certification. This implies that it might be fruitful to seek to resolve this issue in a federation-wide fashion. This could be done by community wide effort to develop training and certification components as part of the caBIG™ program.

### A central auditing authority is a necessity

#### All data sets dealing with human data, whether de-identified, limited, of fully identified, should be subject to the same auditing requirements

Auditing should be performed in the same manner and level for all data sets dealing with human data. This includes de-identified, limited data sets, and identified data. The same auditing data should be captured regardless of risk level of the data itself.

From the perspective of the policy and compliance personnel interviewed the data support the requirement for a specific body to oversee auditing. This group should be developed and empowered to define compliance standards with policies, and to enforce these standards via an accreditation process. Specific auditing functions this central group would be charged with overseeing include both technical and non-technical components, and consist of policy review, adherence to agreements, adherence to technical procedure and technical security architecture, adherence to data release only through protocol, incident aggregation, incident analysis, and communication of audit data back to the member institution. The audit group should provide a statement of compliance or non-compliance with key policies and procedures for each member institution.

#### Specific tooling to support the auditing functions is needed

Given the need for some form of centralized auditing support, the technical considerations are not trivial. Every institution involved in a federation must have technology support for the relevant security and privacy logs. However, coherent global audit requires efforts to standardize security data elements and communication protocols. Otherwise, the power of the auditing capability is reduced to simply a set of local audits that may not appropriately address systematic and end to end security and privacy issues. Consequently, specific tooling is needed to support both the centralized auditing functions proposed for the governing body, and the specific data required for trust development at the individual institutions. Interviews suggest a preference for auditing at the individual record level even when dealing with de-identified data. Local groups need assurance that the remote audit data is being properly maintained, that it has an acceptable retention period, and that it is available to them for inspection on demand.

#### A Two-protocol Mode for Data Exchange is accepted by interview participants

An important finding of this research was that, in general, participants accepted the idea of a two-protocol mode for data exchange for de-identified data. In this model, both the repository owner and the investigator (who may be at different institutions) may have IRB protocols from their respective institutions. The relationship between parties can be emergent and does not need to be specified in advance, as long as all understand and agree to this approach. This approach mirrors (at the data exchange level) the governance structure of successful identity federations such as InCommon, SAFE, and the Liberty Foundation.

#### Critical IRB issues remain that must be resolved

This study raised several important IRB issues that should be clarified by development of community consensus including:

• The manner in which undefined prospective research involving data and tissue repositories will be consented and handled.

• How data use and confidentiality agreements can be established between participant organizations and individual investigators in a scalable fashion.

• The development of common consent forms acceptable to all IRBs participating in a federation.

In particular, the development of a common consent form would greatly facilitate multi-institutional prospective research projects, but would require strong leadership and involvement of the individual IRBs, potentially including face-to-face meetings of IRB representatives. Participants suggested that this is important work where NIH leadership is needed.

#### Limitations

Limitations of this study include potential selection bias, and difficulty of participants describing risks of a novel and unfamiliar technology.

Participants in this study were limited to stakeholders at cancer centers who had already agreed to participate in caBIG – a federated biomedical data grid. Thus, it is likely that the institutions from which our stakeholders were selected, have already bought in to the concept of data federation. Participants from these institutions may be more accepting of the basic premises of federation than participants drawn from centers who are not participating in the caBIG.

Throughout the interviews, we found that participants had difficulty with some questions related to assessing risks for such a technology as novel as a federated grid system. Privacy and security requirements are typically considered only on the scale of an individual institution or known business partners. Envisioning a world where security must be managed across multiple, unknown partners is a daunting task for many participants. Thus, further efforts to gather security and privacy requirements should be undertaken as federated systems emerge.

Although the number of participants in this study was relatively small, we note that the sample includes most stakeholders at five of the fifty existing NCI designated cancer centers, representing a 10% sample of the institutions. Given the detailed nature of the interview instrument, the sample was deemed sufficient for the purposes of requirements gathering. However, it is entirely possible that other security and privacy concerns and requirements may exist which were not uncovered in these interviews.

Finally, this work represents a survey study prior to actual design and implementation of data exchange systems. The course suggested holds substantial sociologic challenges in the reorganization of regulatory practices across multiple institutions. This work will need to be further validated by comparison to functioning systems at a future date.

#### Further Observations

The recently created NIH Genome-Wide Association Studies (GWAS) program [37] and the data-sharing policies emerging from it represent an interesting development. The GWAS data sharing mechanisms for the dbGAP database are an example of a new program that has been constructed along the general lines of the framework discussed in this paper. Data submission to dbGAP requires pre certification by institutional officials in advance of data submission and the pre certification must be part of data sharing plan submitted with grant applications. Data quality, security and privacy are maintained to certain standards and there are guidelines in place for both the data repository at the National Library of Medicine, as well as the research groups submitting the data. Access to the database and use of data extracted from it requires review by a data-access committee and a formally submitted data-use certification agreement that must be signed by institutional officials. Data distribution is bound by additional constraints, including publication embargo of developed results. Finally, there are mechanisms in place to audit and review appropriate data access and use.

The GWAS agreement uses a strong central governing structure and places responsibility for adherence to the terms of the data sharing and acceptable use agreements on the institutions. This model is similar to existing animal and human subject protection mechanisms in that: (a) an institutional-level commitment to protection and compliance is required, and (b) the agreements are specific to the resource. The GWAS policy effectively lays out resource-specific risk-assessment and risk-mitigation plans to be carried out by all parties. In essence, all parties have agreed to meet uniform minimum requirements and standards, overseen by a common governance framework to protect each other and third parties from risk. This framework has many of the elements discussed in this paper, with the minor variation that the NIH has chosen to maintain dbGAP as a centralized resource, rather than deploying a distributed or federated technology model. Table 1 of Piwowar [38] summarizes possible classifications and the tradeoffs involved in selecting a centralized or competing data sharing model.

The present study suggested, and the structure of the GWAS repository confirms, that formal, advance risk-mitigation and institutional sign-off mechanisms should be in place before sensitive data are collected and shared through collaborative resources. There is high scientific value in collecting and sharing very large and highly distributed data sets, but there is also high risk associated with maintaining sensitive information in a distributed, collaboratively operated information resource. These joint pressures are driving the need to develop more detailed, and more specific social mechanisms to permit collaborative research while maintaining individual accountability.

These trends are likely to continue and it would not be surprising if institutions soon begin to deploy information security oversight committees, designed and operated in a manner similar to institutional review boards and animal care and use committees where the security details of individual proposed research projects are reviewed and approved in the context of general guidelines and applicable laws and regulations, all supported by appropriate local infrastructure.

## Conclusion

This study identified and explored security and privacy requirements for large-scale federated biomedical data sharing initiatives. Study data suggest many areas of consensus among a sample population of stake-holders, and also indicate areas where there is greater variability. It also elucidates stakeholder opinions in the areas of governance, identity provisioning, auditing, honest brokering and research training certification. Based on these opinions, the authors propose an initial set of security and privacy requirements for an emerging federation. These requirements are currently being used as a model for the development of the caBIG data sharing and security framework (DSSF). These requirements also represent a general framework that can be used to inform the development of other large multi-institutional data sharing consortium. The findings, as well as the experiences of other large scale data sharing initiatives suggest that data sharing mechanisms will increasingly require strong central governance, and institutional commitment to the security procedures and policies of these organizations. It may well be that we are seeing the development of risk mitigation strategies and institutional sign off requirements on a resource-specific basis. The implications for applied technology and biomedical informatics practitioners will be the need to develop new applications and knowledge infrastructure to support the processes of security, privacy, and trust management in the regulated environment.

## Competing interests

The authors declare that they have no competing interests.

## Authors' contributions

RSC, FJM, RJR, and WAW developed the research protocol and ran the focus groups that developed the survey instruments. RSC conducted the survey interviews and performed the qualitative and quantitative analyses. FJM, RJR, and WAW reviewed the analysis and performed technical review and implications. RSC and FJM drafted the manuscript, and all other authors were involved in critical review and revision of the manuscript. All authors have given final approval of the version to be published.

## Pre-publication history

The pre-publication history for this paper can be accessed here:

http://www.biomedcentral.com/1472-6947/9/31/prepub

## Supplementary Material

Additional File 1 **Structured interview instruments**. This file contains the four interview instruments that were used in the study. The questions for these instruments were developed using a team-based approach as described in the methods section of the paper. Click here for file
