# Supplementary material for: Security and privacy requirements for a multi-institutional cancer research data grid: an interview-based study
Source: BMC Med Inform Decis Mak. 2009 Jun 15;9:31. doi: 10.1186/1472-6947-9-31 (PMC2709611; doi:10.1186/1472-6947-9-31)
Supplement: Additional File 1 — Structured interview instruments. This file contains the four interview instruments that were used in the study. The questions for these instruments were developed using a team-based approach as described in the methods section of the paper. [file 1472-6947-9-31-S1.doc]

**Structured Interview Instruments**

The following four instruments were constructed by a team of thirty-eight individuals representing a wide spectrum of experts and stakeholders from US Cancer Centers. Participants developed issues that might pose problems, particularly those where we expected significant variation among cancer centers. Issues were collected into a master list and sorted into the four emergent categories of: (1) Locus of control/decision making, (2) De-identification and IRB Policy, (3) Authentication and Authorization, and (4) Consenting. Participants then divided into four breakout groups, one for each of these major themes, and constructed scenarios and draft interview questions designed to elicit information during the interviews. All scenarios used caTIES as the example system. Participants met at the end of the day to critique the resulting scenarios. The authors then edited the interview scenarios to ensure adequate coverage of the issues, improve the understandability and simplicity of the interview questions, and match interview questions to organizational roles of interviewees. The resulting draft instruments presented below were reviewed by all meeting participants, and modified in three subsequent rounds of editing and draft revisions. The instruments contain a total of 87 questions, which is arrived at by counting both the major questions (as numbered below) and their subcomponents where applicable.

**Scenario 1 – Identification of local organizational environment, stakeholders, and decision-making processes**

**Target respondents: IRB member, Security Officer, HIPAA Compliance Officer, Office of Research**

Your institution is interested in participating in a new NCI initiative – the Cancer Bioinformatics Grid (caBIG). This multi-institutional project will provide researchers with access to data and computer applications that reside in multiple collaborating cancer centers across the country. Several investigators who are interested in adopting some of the caBIG tools have set up an informational meeting among potential stakeholders, including IRB officials, security officers, HIPAA compliance officers, and university/hospital counsel. During this meeting, the investigators are interested in getting a better understanding of your institution’s position on data sharing and privacy, particularly existing organizational structures and decision-making processes. They pose the following questions, and ask that you answer them based on your knowledge of your own institution.

1. Do you have a system(s) under which a disinterested party provides de-identified data for research purposes (an “honest broker system”)? If so, please describe how your honest broker system is set up (feel free to attach any supporting documents). Specifically, please explain how it is situated organizationally within your institution.
2. Is your process for de-identification capable of re-identifying the subject consistent with IRB and HIPAA requirements? How is this accomplished?
3. In what situation, if any, do you think most people/institutions would want to have a governing body at the multi-institutional level? Why? In your opinion, what particular functions will such a governing body need to take on?
4. Do you distinguish between data “steward” and data “owner”? What are the respective roles and responsibilities of data stewards and data owners?
5. What specifications are required for meeting the “HIPAA safe harbor” standard of de-identification of patient/human subject data so that it can be used for research? How about for the “HIPAA limited data set” approach? What person or body within your institution sets these standards?
6. Is the local IRB and security officer willing to take on the responsibility of indemnifying the service provider? If so, under what conditions?

During the meeting, the researchers identified Identity Provisioning as a particularly concerning issue. It is expected that thousands of individuals at over 100 institutions will have access to at least some of the data exposed to this network. There are concerns about how to best administer identities in such a network.

1. Who should be responsible for creating identities and authorization?
2. What type of federated credential would you be willing to accept to identify an individual outside of your institution for the purposes of granting authentication to access data from your institution?
3. What information do we need to collect about users that will provide your institution with enough details to make decisions about granting access?
4. If you found out that Institution B was not following required procedures systematically, would you want to discontinue working with Institution B?
5. What would your institution consider acceptable monitoring of credentialing? Who would be monitoring credentials at your institution?
6. What assurances are needed by one institution to know that an individual applying for the credential at another institution is who they say they are?

The researchers are also concerned about how to make decisions about who can access the data and under what conditions. They ask for your guidance regarding the following:

1. What are the situations where you would decide to turn off access to data, and who makes that decision?
2. What would be required for you to authorize a “step-up” request, i.e., a request for access to data that carries a higher risk of re-identification of the data? (Note: a “step up” request asks for access to data that has a higher risk of its “de-identification” being compromised. For example, queries involving rare diagnoses that may return a single or few results.)
3. What information about a user and certifications (e.g., HIPAA training) do you normally require for data use?
4. Is there additional information you require due to the nature of this multi-institutional project?
5. Of those items you are required to receive, which would you be willing to accept from a third party versus those you must maintain and verify locally?
6. What aspects of the authorization process must you be in control of in order to participate in the project?
7. Who is responsible at your institution for tracking disclosures of PHI? Describe your mechanisms for dealing with such a disclosure.

**Scenario 2 – De-identification**

**Target respondents: IRB members**

Dr. Reddy, director of translational research at your institution calls you to tell you that they wish to become a part of the caTIES network. They would like to become a data service provider, meaning that they would contribute de-identified surgical pathology records to be available for researchers at your institution and other institutions that are part of this network of cancer centers.

1. What kind of IRB protocol would likely be required (e.g. full review, expedited, exempt, not human subjects research)?
2. Does this activity qualify as human subjects research? If you need more information to make this determination, what additional questions would you want to ask?
3. If this is considered human subjects research, what specific risks (particular harms) are you most concerned about for the human subjects of the protocol?
4. Are there specific standards that must be met by other institutions that participate in this network in order for you to approve the protocol?
5. Does it matter whether the data is free-text (e.g. in a pathology report)with no primary identifiers (anonimized), as opposed to structured data (discrete data elements entered into fields)? How would this change your analysis?
6. Does your institution have an approved process for de-identification?

A few weeks later, you receive a protocol from Dr. Reddy which includes additional details. The software application accesses records that are de-identified using an automated tool. Records are reviewed and the occurrence of patient identifiers is extremely low but can occur. No other method is practical as the number of records is in the hundreds of thousands. There are additional quality assurance checks of anonymized records and the algorithm can catch and remove most identifiers.

1. Under what conditions would you approve this protocol? What concerns or questions would you have? What additional information or assurances would you need?
2. What, if anything, would you want to know about the anonymization process (algorithm, operation, implementation, oversight, auditibility)?
3. Does it matter whether the data is retrospective (previously collected) or prospective (to be collected after the study is approved)? How would this change your analysis?
4. The occurrence of identifiers in the documents is rare. However, there is some risk, which varies depending on the data element. Could you estimate an acceptable risk for each of the elements below (e.g. <1/1000, <1/10,000)

- Patient initials (e.g. JKS)
- Sample accession number (e.g. UD-23123)
- Doctor’s initials (e.g. DSS)
- Hospital Name
- City, Town or other location

1. What, if any, role does your IRB have in ensuring that your institution complies with the HIPAA Privacy Rule?
2. Does your institution have a more specific definition or implementation of de-identification/anonymization processes (for example, is removal of data elements in addition to those enumerated by HIPAA’s “safe harbor” required)?
3. What specific data fields if any do you feel are most likely to increase the probability of successful re-identification? How should such fields be handled?

Dr Reddy’s approved IRB protocol to be a data provider to the network is general in that it states that he will provide data to all institutions associated with caBIG. A new US institution (Institution X) joins caBIG and the caTIES network. Institution X was not a member of the caTIES network when Dr. Reddy’s IRB protocol at your institution was granted. Therefore Dr. Reddy does not specifically know anything about the researchers who will be accessing data generated by your institution.

1. How would the entry of Institution X affect your analysis regarding de-identification?
2. What assurances would you need in place to continue to approve Dr. Reddy’s protocol?

A number of participating institutions have felt the need for trust agreements that would formalize the credentialing of users, and security policies and procedures to be followed by members of the network.

1. For each element below, please comment on whether a trust agreement between institutions which included this element would affect your analysis and decision making for the scenario above:
   1. Level of integrity protections (encryption, physical security, etc).
   2. Statement that users will not attempt to re-identify previously de-identified data
   3. Statement that users will not attempt to use data for purposes other than those allowed by the trust agreement
   4. Liability allocation
   5. Indemnification (not allowed for federal; usually not allowed for state)
   6. Assurance and/or certification that staff with access to the data will receive training, including on privacy and security
   7. Agreement to participate in defined security incident response policies and procedures
   8. Penalties for breaching the terms of the agreement (please specify)
2. What other agreements would you like to see in place in the trust agreements?

Dr. Reddy contacts you again. Many researchers would like to use the caTIES system to determine the number of specimens that are available to use as preliminary findings in their grant applications. Also, he has been contacted by at least one person from outside the University who is interested in using the system to find out what tumors are common and uncommon. Dr. Reddy would now like to provide aggregate data to the general public in the form of histograms based on discrete intervals. For example, he would like to be able to show the number of Hepatocellular carcinomas over the last twenty years, for patients by age in 5 year increments. The system will not return anything more specific than this aggregated data.

1. Do you consider this human subjects research? Under what conditions?
2. If this is considered human subjects research, what specific risks (particular harms) are you most concerned about for the human subjects of the protocol?
3. Given that the system is available for query, it would be possible to maliciously use the system to determine patient information. For example, if you know that an 89-year-old patient was diagnosed with a brain tumor in the summer of 2004, and there was only one 89-year-old patient diagnosed with a brain tumor that summer – it might be possible to identify the patient. The number of records in an aggregate data set can be considered the “bin size”. Do you have any specific recommendations about the minimum bin size for aggregate data?

Dr. Reddy calls you again a year later. His good friend in the UK would like to become a member of the caTIES network as well, and UK researchers would then be able to access the de-identified reports at your institution.

1. What do you want to see from a foreign partner to approve the process going forward?
2. Are there specific international partnerships that you view as problematic? Why?

**Scenario 3 – Auditing**

**Target respondents: Security Officer, HIPAA Compliance Officer, and Office of Research**

You receive a telephone call from your Institutional Review Board. A recently submitted protocol has generated some discussion, and your input is needed. A researcher at your institution has a collection of pathology reports and DNA microarray analysis reports that they would like to share with other researchers. The project will be quite novel, because it proposes a network of computers that will permit investigators at any single institution to access data from all other institutions. The data that will be available includes de-identified data derived from free-text pathology reports and DNA microarrays, but does not contain Protected Health Information (PHI) as defined by HIPAA. One of the novel aspects of the project is that there will not be a central organization that administers all users; rather it is expected that each institution will administer its own users and all institutions will agree to a set of guiding principles and practices. Because of the unique aspects of this proposal you have been asked to evaluate the project from a security compliance point-of-view.

1. What access-control and usage auditing activities will be required for you to grant permission?
2. What record keeping and accountability will you expect? (General institutional level trust agreement, Protocol-specific institutional agreement, Individual appropriate-use agreements by each user, Other)
3. Will you require that an audit trail be maintained for data use? At what level of detail? (Session, Data set, Data record, Data element)
4. What auditing information would be required? (Who has accessed the data, who can access the data, who granted access to the data, on what basis, and when?)
5. Who should be responsible for generating audit data?
6. Who should be responsible for managing audit data?
7. Do you have a required time interval for maintaining an access log?
8. What different access and audit requirements will apply for the data to be shared as de-identified data, limited data set data, fully identified data?

The researcher notes that some users may wish to access the data via workflow tools that will pass the data through several analytical tools en route to the user. (i.e., these analytical tools may reside anywhere, not just at the originally defined collaborating institutions). In the course of this process, the data may be altered or aggregated according to a specific research protocol.

1. How does this impact your analysis?
2. Are there additional requirements?
3. Do you care where the tools reside?
4. Do you require trust agreements with the providers of the analytical tools?
5. Do you require an audit trail of the data passage through the analytical tools?
6. What kind of progress report will you expect for data usage?

After modifications to the project based on your concerns, the project has been approved by the IRB and all other required entities, and data sharing begins. Six months after the project begins, the researcher at your institution comes to see you. A new question has emerged among the user group for the project. A researcher working in private industry wishes to access the data anonymously, due to the need to protect intellectual property of the corporation doing the research.

1. What concerns do you have about anonymous access to the data?
2. Would this violate any required processes or practices at your institution? If so, please describe.

One year later, there are now twenty participating institutions. A security breach occurs at one of the participating institutions. An unauthorized user has gained access to data at multiple institutions, potentially including yours. The incident is reported to you.

1. Describe precisely what you will you need to know about the breach.
2. What requirements do you have for reporting the incident within your institution?
3. Are there special requirements that may arise because of the inter-institutional aspect of the incident?
4. Are there external entities that would require notification? Which entities? Under what conditions must they be notified?

**Scenario 4 – Prospective Research Consenting**

**Target respondents: IRB member, Office of Research**

You are being asked to evaluate a new IRB proposal for a prospective research project using a collaborative grid computing platform. Although you have previously evaluated a protocol that used the same infrastructure, this project is different because of the prospective nature of the work. The proposed project involves both (1) accruing patients into the prospective study so that data and tissue collected could be used by other institutions, and (2) accessing data from external institutions. You have been asked to specifically evaluate the patient consent aspect of the project, to determine whether it meets the requirements of your institution. In further evaluating the protocol, it becomes clear that this project will support numerous individual research projects from investigators at multiple institutions, many of which will not be developed at the time that the protocol is submitted.

1. What elements are needed in the consent form (hereafter “consent items”)? Can you provide a consent form used in another research project at your institution that could be used as a starting-point for the project described?
2. With regard to the issue of undefined future research, please address the scope of the consent. Must the consent form be specific to an individual protocol, or can a more general consent form be used to cover all of the individual projects that will arise?
3. What are the local consent items that might supersede the project level consent items?
4. Is there a hierarchy of consent forms? If a patient consents to share data in caBIG and then consents to participate in local research only, does one take precedence over the other?
5. Will you accept use of an approved IRB consent form developed at another institution, and under what circumstances?
6. Does the development of the research repository need to be articulated in the consent form? Would the PI need to be identified?
7. Do we have the legal right to share patients’ consented data or tissues after their death? How long is the patient’s consent valid?
8. Under what situation(s) would you require investigators to re-consent patients who have previously agreed to participate in the network?
9. What additional or new requirements would apply to individual projects once general consent mechanisms were in place?
